# Frequent occurrence and predicted functions of tRNAs with 4-base-pair anticodon stems in bacteria: extended superwobble hypothesis

**DOI:** 10.1093/nar/gkag327

**Published:** 2026-04-20

**Authors:** Fadel Fakih, Satish Nandipati, Ambar Kachale, Jiří Heller, Jakub Žváček, Filip Brázdovič, Nawal Al-Chamy, Pragya Tripathi, Zdeněk Paris, Leoš Shivaya Valášek, Michal H Kolář, Vyacheslav Yurchenko, Marek Eliáš, Eugene V Koonin, Julius Lukeš, Anzhelika Butenko

**Affiliations:** Institute of Parasitology, Biology Centre, Czech Academy of Sciences, 37005 České Budějovice (Budweis), Czechia; Faculty of Sciences, University of South Bohemia, 37005 České Budějovice (Budweis), Czechia; Institute of Parasitology, Biology Centre, Czech Academy of Sciences, 37005 České Budějovice (Budweis), Czechia; Faculty of Sciences, University of South Bohemia, 37005 České Budějovice (Budweis), Czechia; Institute of Parasitology, Biology Centre, Czech Academy of Sciences, 37005 České Budějovice (Budweis), Czechia; Faculty of Sciences, University of South Bohemia, 37005 České Budějovice (Budweis), Czechia; Institute of Parasitology, Biology Centre, Czech Academy of Sciences, 37005 České Budějovice (Budweis), Czechia; Department of Physical Chemistry, University of Chemistry and Technology, 16628 Prague, Czechia; Institute of Microbiology, Czech Academy of Sciences, 14220 Prague, Czechia; Institute of Microbiology, Czech Academy of Sciences, 14220 Prague, Czechia; Institute of Parasitology, Biology Centre, Czech Academy of Sciences, 37005 České Budějovice (Budweis), Czechia; Institute of Parasitology, Biology Centre, Czech Academy of Sciences, 37005 České Budějovice (Budweis), Czechia; Faculty of Sciences, University of South Bohemia, 37005 České Budějovice (Budweis), Czechia; Institute of Microbiology, Czech Academy of Sciences, 14220 Prague, Czechia; Department of Physical Chemistry, University of Chemistry and Technology, 16628 Prague, Czechia; Department of Biology and Ecology, Faculty of Science, University of Ostrava, 71000 Ostrava, Czechia; Department of Biology and Ecology, Faculty of Science, University of Ostrava, 71000 Ostrava, Czechia; Computational Biology Branch, Division of Intramural Research, National Library of Medicine, National Institutes of Health, Bethesda, MD 20894, United States; Institute of Parasitology, Biology Centre, Czech Academy of Sciences, 37005 České Budějovice (Budweis), Czechia; Faculty of Sciences, University of South Bohemia, 37005 České Budějovice (Budweis), Czechia; Institute of Parasitology, Biology Centre, Czech Academy of Sciences, 37005 České Budějovice (Budweis), Czechia; Faculty of Sciences, University of South Bohemia, 37005 České Budějovice (Budweis), Czechia; Department of Biology and Ecology, Faculty of Science, University of Ostrava, 71000 Ostrava, Czechia

## Abstract

Recently, a tRNA^Trp^_CCA_ with a 4-base-pair (bp) anticodon stem (AS) was shown to efficiently recognize a near-cognate UGA codon in unicellular eukaryotes, such as some trypanosomatids and ciliates, thereby representing a novel codon reassignment mechanism. To determine whether this mechanism also evolved in bacteria, we analysed a dataset of 42 109 genomes, including previously reported cases of stop-to-tryptophan UGA reassignment and a newly identified instance in the phylum Patescibacteriota. We show that the 4-bp AS tRNA^Trp^ species are present across diverse bacteria and in some cases likely function in decoding in-frame UGA codons. Most notable is the endosymbiotic bacterium *Candidatus* Zinderia insecticola, which contains only the near-cognate 4-bp AS tRNA^Trp^_CCA_, while lacking both canonical 5-bp AS tRNA^Trp^_CCA_ and a tRNA^Trp^_UCA_. The secondary structure of this 4-bp AS tRNA^Trp^ resembles that of its eukaryotic counterpart, suggesting convergent evolution. We experimentally confirmed the UGA readthrough capacity of 4-bp AS tRNA^Trp^_CCA_ in *Escherichia coli*, and applied molecular dynamics simulations to suggest the underlying mechanism. Furthermore, we tested several predictions based on accepting the previously excluded possibility of C:A base pairing at the 3rd codon position. These findings provide new insights into the structural diversity of transfer RNAs (tRNAs) and expand our understanding of genetic code evolution.

## Graphical Abstract

**Graphical Abstract:**
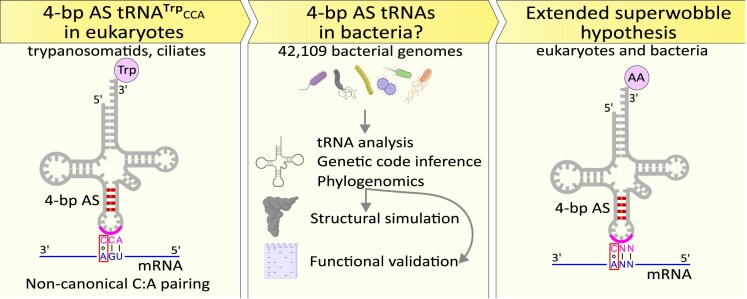

## Introduction

Transfer RNAs (tRNAs) are key molecules in the translation process, responsible for delivering amino acids to the ribosomes, where they are incorporated into the growing polypeptide chain. Moreover, tRNAs are now also recognized as participants in translational control, transcription, splicing, retroelement regulation, nutrient sensing, and apoptosis, as well as carriers of intermediates in several biosynthetic pathways [1]. tRNAs are encoded by genes of variable structural organization, including split and circularly permuted ones [2].

Most mature tRNAs in bacteria, archaea, and eukaryotes adopt a highly conserved ‘cloverleaf' secondary structure consisting of the D, anticodon, variable, T and acceptor arms, and an L-shaped tertiary fold [3–5]. Functional tRNAs typically range from 70 to 100 nucleotides (nt) in length, with variations attributed mainly to differences in the length of the variable and D arms, whereas the other parts of tRNA molecules are highly conserved, with only rare exceptions [6, 7]. Substantial deviations in the tRNA secondary structure have been documented in mitochondria, where the helical regions may vary in length or even be missing, with some of these alterations being reflected at the level of the tertiary structure [8]. Notably, mitochondrial tRNAs exhibit deviations in the number of paired bases within the anticodon stem (AS), demonstrating up to 10-bp AS [8]. The relationship between deviations in the length of structural elements within tRNAs and their functions remains largely unclear [6]. The tRNAs that depart from the structural consensus are generally perceived to be unable to function in translation [3]. The decoding capacity of tRNAs can be affected by several different mechanisms including post-transcriptional modifications, to which ~12% of nucleotides in a typical tRNA are subjected [9, 10].

A minimum set of 32 tRNAs is required to decode 61 sense codons of the genetic code according to the Crick’s ‘wobble rule’ [11, 12]. Under these premises, non-Watson–Crick base pairing, in particular, G:U, U:G, and I (inosine):A/C/U can occur at the wobble (3rd) position of the codon–anticodon minihelix, allowing for flexibility in translation [12]. The wobble rule was later expanded after the discovery that tRNAs with an unmodified U in the wobble position of the anticodon can pair with any of the four bases at the third position of the codon via a phenomenon called ‘superwobbling’ [13]. More recently, the T-arm sequence in *Mycoplasma mycoides* tRNA^Gly^ was identified as a novel pivotal element facilitating superwobbling [14]. Overall, the extension of possible codon–anticodon interactions through wobble and superwobble rules provides a mechanism, by which some bacterial and many organellar genomes, which lack a complete tRNA set, can achieve recognition of all four nucleotides at the third codon position [11].

The flexibility of tRNA decoding has been largely attributed to modifications in the anticodon loop, primarily at the wobble position 34. For example, in human mitochondria, 5-formylcytidine (f^5^C) at this position permits decoding of both AUG and AUA codons by tRNA^Met^_CAU_ [15]. In bacteria, as well as in plant plastids and mitochondria, tRNA^Ile^_CAU_ with lysidine (k^2^C) or its derivative 2-aminovaleramididine (ava^2^C) at position 34 favours AUA decoding as isoleucine, while restricting recognition of AUG [16–20]. A prominent group of wobble-base modifications are the xo^5^U derivatives, including the 5-carboxymethoxyuridine (cmo^5^U, also known as uridine-5-oxy acetic acid). These xo^5^U type modifications facilitate non-Watson–Crick base pairing with guanosine and pyrimidines at the third positions of codons, thereby broadening the decoding range of the associated tRNAs [21–23]. Additional tRNA modifications that enable decoding of the near-cognate stop codons via non-canonical base pairing, while largely maintaining the ability of tRNAs to decode cognate or near-cognate sense codons, were recently reported [10]. Codon recognition by tRNAs can also be expanded by adenosine-to-inosine (A-to-I) edits that in both bacterial and eukaryotic tRNAs enable I^34^ to read codons with A, C, and U bases [24]. Moreover, cytidine-to-uridine (C-to-U) editing in kinetoplastid mitochondria generates a tRNA^Trp^ variant capable of decoding UGA as tryptophan [25, 26].

Not surprisingly, modifications and/or specific point mutations can also alter tRNA’s ability to mediate translational readthrough of stop codons [27]. The so-called suppressor tRNAs with the anticodon mutated such as to enable recognition of stop codons as sense codons (thus, suppressing phenotypes resulting from the occurrence of in-frame termination codons in other genes) have been documented in viruses, bacteria, archaea, and eukaryotes, and are the most common molecular solution for decoding UGA, UAA, or UAG in organisms with an altered genetic code featuring stop-to-sense codon reassignments [28–32]. Point mutations occurring outside the anticodon, e.g. Hirsh mutation G24A and A9C, can also modulate the effectiveness of nonsense suppression [33, 34].

We have previously demonstrated that a relatively minor structural change in tRNA^Trp^_CCA_ of the trypanosomatid *Blastocrithidia nonstop* and the ciliate *Condylostoma magnum*, namely ‘shortening’ of its canonical 5-bp AS to 4-bp by unpinning the top base pair (26C·42U), dramatically increased UGA stop codon readthrough in the reporter systems in *Trypanosoma brucei* and *Saccharomyces cerevisiae* [35, 36]. Here, ‘shortening’ refers to the disruption of a Watson–Crick base pair. This shortening does not imply collapse of the AS, as one might infer from conventional 2D secondary structure diagrams, but rather a subtle and coordinated modulation of the tRNA structure and dynamics, often in a non-local manner as discussed below.

Importantly, this 4-bp-long AS tRNA^Trp^_CCA_ is a natural and the only form of tRNA^Trp^_CCA_ in *B. nonstop* and *C. magnum*, i.e. organisms with UGA reassigned as a tryptophan codon, in the latter species in a homonymous configuration preserving the ancestral function of UGA as stop when occurring at the coding sequence ends [32, 35, 37, 38]. The tRNA^Trp^_CCA_ with an identical structure, i.e. carrying the 4 bp-long AS, was subsequently identified in the genomes of other eukaryotes with reassigned UGA (fully or homonymously) to tryptophan in their nuclear genomes, namely, ciliates of the genera *Blepharisma* and *Loxodes* [39], other species of the genus *Blastocrithidia* [40], and the dinoflagellate *Amoebophrya* sp. ex *Karlodinium veneficum* [41]. Moreover, the expected ‘mutant’ cytosolic tRNA^Trp^_UCA_ (fully cognate to UGA) could not be identified in *Amoebophrya* sp. ex *Karlodinium veneficum, Blastocrithidia* spp., and *C. magnum* [35, 37, 40, 42]. Functional association of 4-bp AS tRNA^Trp^_CCA_ with the UGA readthrough is further strengthened by the absence of this ‘unpinned’ tRNA in the genomes of ciliates *Stentor* and *Fabrea* (close relatives of *C. magnum*), both of which use UGA solely as a termination codon [39].

In light of these unexpected findings, the results of an early mutational screening of suppressor tRNAs in *Escherichia coli*, which linked 4 bp-long AS with readthrough of the UAA stop codon, come to prominence. They demonstrated a normally disfavoured C:A base pairing at the tRNA wobble position [43], thereby further expanding the flexibility of decoding by tRNA. Since 1980s, bacteria that interpret in-frame UGA codon as an amino acid are known and new cases are being reported [32, 44]. Point mutations in the anticodon making tRNAs fully cognate to the respective stop codons appear to be the main mechanism of translational readthrough of in-frame stop codons in bacteria. However, a recent report identified a 4-bp AS *tRNA^Trp^_CCA_* gene in a bacterial endosymbiont possibly hosted by dinoflagellates, providing an alternative putative mechanistic explanation for the stop-to-tryptophan UGA reassignment in this bacterium, where unpinning of the top base pair in the AS rather than anticodon mutation enables translational UGA readthrough [45].

Collectively, these data indicate that structural alterations of the AS can affect tRNA codon specificity in eukaryotes and bacteria, precluding prediction of codon specificity based solely on the Watson–Crick base pairing [46]. Although specific mutations at positions distant from the anticodon loop (AL) are known to change tRNA’s specificity [27], the alteration of codon recognition properties via the above-described AS unpinning remains unprecedented.

Here, we analysed tRNAs encoded in the bacterial genomes where, in addition to eukaryotes, multiple instances of stop-to-tryptophan UGA reassignment have been identified (‘Graphical abstract’ section). Our results showed a surprisingly frequent occurrence of tRNAs with 4-bp-long AS that is not limited to tRNA^Trp^. We also demonstrate that the 4-bp AS *tRNA^Trp^_CCA_* genes are naturally present in some bacterial genomes exhibiting stop-to-tryptophan UGA reassignment and could mediate decoding of UGA. By introducing an engineered 4-bp AS tRNA^Trp^_CCA_ into *E. coli*, we show that unpinning of the top base pair in the tRNA^Trp^_CCA_ AS indeed enables the UGA readthrough in this model bacterium.

## Materials and methods

### Identification of tRNAs in bacterial genome assemblies

Bacterial genome assemblies and the associated metadata (e.g. taxonomic affiliation, information on assembly size, GC content, contamination level, etc.) were downloaded from the Genome Taxonomy Database (GTDB) release 214.1 [47]. Genomes designated as representative for each species in the GTDB (typically those of a type strain with high-quality assembly) and demonstrating ≤1% contamination were retained for further analysis. Since a new version of the GTDB was released during the preparation of this manuscript, the taxonomic data have been updated to reflect the changes introduced in the GTDB release 226. Additionally, five genomes of endosymbiotic bacteria absent from the GTDB (*Ca*. Carsonella ruddii, *Ca*. Hodgkinia cicadicola, *Ca*. Karelsulcia muelleri, *Ca*. Nasuia deltocephalinicola, *Ca*. Pinguicoccus supinus), all exhibiting ≤1% of contamination, were downloaded from NCBI. For these genomes, contamination was assessed using CheckM v.1.2.2 [48], with the marker lineage set to Bacteria (UID2495). A total of 42 109 genome assemblies were retained for further analysis (Supplementary Table S1).

The tRNAs genes were predicted using the standalone version of tRNAscan-SE with the ‘-B’ (bacteria) option, and other parameters left as default [49]. Identified genes labelled by the software as putative pseudogenes and those of undetermined type (tRNA type column value ‘undet’ and/or anticodon column value ‘NNN, NNA, TGR, GTN, or CCR’) were excluded from subsequent analyses. Only tRNAs featuring anticodons within positions 33–37 were retained for the analyses and categorized based on their AS length, which was binned into 5-bp, 4-bp, and ‘other’ categories. When categorizing tRNAs with bulges (i.e. unpaired positions) in the AS, the total number of nucleotides between and including the outermost paired nucleotides, was considered. For example, a tRNA with four paired and one unpaired nucleotide in between in its AS was classified as a 5-bp AS molecule. The categorization of the tRNA structures in the output files of tRNAscan-SE was carried out using an in-house Python script available upon request. A separate category of ‘*Blastocrithidia*-like tRNA^Trp^_CCA_’ was defined as comprising tRNAs with 4-bp AS, 7-nt-long AL, and lacking A9C and G24A mutations. tRNA^Trp^_CCA_ were scanned for these features using a regular expression search in the output files of tRNAscan-SE containing secondary structure predictions. For some analyses, tRNAs were grouped based on their percentage out of all identified tRNAs, with those representing <0.1% of the total number of identified tRNAs designated as ‘low abundance’, and the remaining ones considered ‘high abundance’.

For the analysis of tRNA^Arg^_CCU_ in the genomes with arginine-to-methionine AGG reassignment, six additional genomes (accessions GCA_002404995.1, GCA_002431755.1, GCA_002439645.1, GCA_002436825.1, GCA_002451385.1, GCA_002297105.1 ) were downloaded from NCBI (24 November 2024) and tRNAs were predicted using tRNAscan-SE and ARAGORN v.1.2.41 [50]. The plot demonstrating the correlation between the genome size and the fraction of 4-bp AS tRNAs along with the related statistics (Pearson correlation coefficient and *P*-values) were generated in MS Excel 365 (https://www.microsoft.com/cs-cz/microsoft-365/excel; last accessed: 3 September 2025). For the analysis of the presence of tRNA_UNN_ along with the 4-bp AS tRNA_CNN_ or without (i.e. when only the 5-bp AS tRNA_CNN_ is present), only bacterial genomes possessing at least one *tRNA_CNN_* gene were selected considering each of the 11 amino acids encoded by both NNA and NNG codons (Ala, Arg, Gln, Glu, Gly, Leu, Lys, Pro, Ser, Thr, and Val). Since high fraction of bacterial genomes lacks tRNA^Arg^_UCG_ (regardless of the presence of a 4-bp or 5-bp AS tRNA^Arg^_CCG_), which is explained by the fact that bacteria generally decode the CGA codon with a tRNA^Arg^_ICG_ originating from tRNA^Arg^_ACG_ by post-transcriptional adenine-to-inosine conversion at the first anticodon position, we excluded the tRNA^Arg^_CCG_/tRNA^Arg^_UCG_ pair from this analysis. In addition, the genomes where the meaning of the NNA and NNG codons differs, were excluded from the analysis. Statistical significance of the observed differences was evaluated using the two-sample proportion *Z*-test (https://www.statskingdom.com/121proportion_normal2_0.html; last accessed: 3 September 2025).

To determine whether the percentage of UGA stop codons (out of all identified stop codons) was lower in bacterial genomes encoding exclusively 4-bp AS tRNA^Trp^_CCA_ compared to those encoding only 5-bp AS tRNA, we analysed a dataset of 2677 bacterial genomes containing only the 4-bp AS variant (Supplementary Table S1). For statistical comparison, we selected bacterial orders containing ≥10 genomes that exclusively encoded 4-bp AS tRNA^Trp^_CCA_ and compared them to genomes from the same orders that encoded only the 5-bp AS variant. To determine whether there are statistically significant differences in UGA percentages, the Shapiro–Wilk test was used to assess normality (with the significance level of 0.05). Depending on the distribution, either a Mann–Whitney U test (for non-normal distributions) or an independent *t*-test (for normally distributed data) was applied to assess statistical significance (both with the significance level of 0.05). All tests were performed using Statistics Kingdom web page (https://www.statskingdom.com/121proportion_normal2_0.html; last accessed: 3 September 2025).

### Mapping the distribution of the bacterial tRNA^Trp^_CCA_ variants on the phylogenomic tree

Bacterial phylogenomic reference tree based on the concatenated alignment of 120 proteins was retrieved from the GTDB release 214.1. For visualization purpose, branches corresponding to the species not included in the current dataset were removed from the tree. The tree was visualized using the Interactive Tree of Life (iTOL) web server, and the tRNA mapping was done via the annotation files containing the genome IDs and tRNA variant’s presence/absence data [51].

### Comparison of 4- and 5-bp AS tRNA sequence conservation and synteny analysis

To compare sequence conservation levels between the cohorts of 4- and 5-bp AS *tRNA^Trp^_CCA_* genes, we selected bacterial genomes that contain 4- and 5-bp AS *tRNA^Trp^_CCA_* genes simultaneously. Using a custom Python script employing MAFFT v.7.505 with the L-INS-i algorithm [52] to perform pairwise sequence alignment (available upon request), we obtained pairwise sequence identity values for the sets of 4- and 5-bp AS *tRNA^Trp^_CCA_* genes. Synteny analysis was performed with Easyfig v.2.2.5 with the default settings [53].

### Gene expression analysis

For comparing the expression levels of 4- and 5-bp AS *tRNA^Trp^_CCA_* in the bacterial genomes where the respective genes co-occur, the available RNA-seq data were retrieved from NCBI SRA [54] and European Nucleotide Archive [55]. Data of sufficient coverage were available only for three species: *Amycolatopsis japonica* (RNA-seq read accession: SRX14259790; SRX14259791; SRX14259792), *Bacteroides oleiciplenus* (SRX9506087; SRX9506088), and *Variovorax paradoxus* (SRX7293081; SRX7293082; SRX7293083). RNA-seq reads were adapter- and quality-trimmed using Trimmomatic v.0.39 [56]. The trimming parameters applied were [TruSeq3-PE-2.fa]:2:20:10 LEADING:3 TRAILING:3 SLIDINGWINDOW:4:15. The ‘MINLEN’ parameter was set to 50 and 30 for paired-end and single reads, respectively. In the case of paired-end reads, only those that remained paired after trimming were used for further analyses. Read quality was assessed with FastQC v.0.11.8 before and after trimming [57]. Trimmed Illumina reads were mapped to the genome assemblies using HISAT2 v.2.2.1 [58] with default settings. The read mappings were sorted using SAMtools v.1.13 [59] and visualized in Integrative Genomics Viewer v.2.17.0 with the allele frequency threshold set to 0.3 [60]. TPM (transcripts per million) values for tRNAs were determined using TPMCalculator [61] with the default parameters for single reads and the ‘-p’ option for paired-end reads.

To determine whether *Blastocrithidia-*like *tRNA^Trp^_CCA_* genes are expressed in bacteria, we downloaded all publicly available Illumina RNA-seq datasets for the respective genomes from NCBI SRA and European Nucleotide Archive. Only the genomes from bacteria with defined full species names were used for the analysis: *Campylobacter hepaticus* (accession: SRX4915420; SRX4915421), *Campylobacter jejuni* (SRX15890787; SRX15890788; SRX15890789), *Clostridium ljungdahlii* (SRX3388079), *Peptacetobacter hiranonis* (SRX5713559), *Portiera aleyrodidarum* (SRX849002), and *Romboutsia ilealis* (ERX546147). The analysis was performed as described above.

### Genetic code inference

The inference of genetic codes for the genomes in the bacterial dataset was carried out using Codetta v.2.0 with default parameters [62]. The initial dataset of genome assemblies demonstrating stop-to-tryptophan UGA reassignment was filtered retaining only the genomes satisfying at least one of the following criteria: (i) suppressor *tRNA_UCA_* is present, (ii) predicted Codetta stop-to-tryptophan UGA reassignment supported by ≥100 Pfam consensus columns (Supplementary Table S1). The absence of suppressor *tRNA_UCA_* was confirmed with ARAGORN v.1.2.41.

### Validation of a putative stop-to-tryptophan UGA reassignment in the bacterial family JAKLIH01

In addition to the two genomes of bacteria from the family provisionally named JAKLIH01 in the GTDB release 214.1 used for most of our analyses and demonstrating stop-to-tryptophan reassignment according to the Codetta results (Supplementary Table S1), we incorporated two additional genomes assigned to the same family in the GTDB release 226 (accessions: GCA_028702045.1 and GCA_028691405.1). The GTDB annotations for these genomes appeared to be incorrect, likely due to gene prediction assuming the standard bacterial genetic code. To address this issue, we predicted protein-coding genes using Prodigal v.2.6.3 [63] with the ‘-g 4’ option.

The phylogenetic position of these four JAKLIH01 genomes was initially assessed using the phylogenomic tree available in GTDB release 226. We then selected 27 additional genomes (Supplementary Table S2) representing a well-supported clade (96% bootstrap support) within the order UBA6257, identified as the closest known relatives of JAKLIH01, and used them for comparative analyses. To confirm the monophyly of the family JAKLIH01, we performed inference of orthologous groups of proteins with OrthoFinder v.2.5.5 (with the options -S blast, -T iqtree, and -M msa, and the remaining options left at their default values) [64] on the genome dataset of JAKLIH01 and their closest relatives. Phylogenomic analysis was performed on 71 proteins encoded by single-copy genes present in at least 80% of the genomes using IQ-TREE v.3.0.1 [65] with automatic model selection and 1000 ultrafast bootstrap replicates. Prior to this, protein sequences were aligned using MAFFT v.7.505 with the L-INS-i algorithm and trimmed with trimAl v.1.4.rev15 using the -gt 0.8 option [66]. The resulting tree was visualized with iTOL.

To assess whether UGA serves as a stop codon in addition to encoding tryptophan, we first identified orthogroups that included at least one protein from a genome with the stop-to-tryptophan UGA reassignment. These proteins were temporarily excluded from the corresponding FASTA files, and the remaining protein sequences from the genomes with standard genetic code were aligned using MAFFT v.7.505 with the L-INS-i algorithm. Alignments showing no gaps within the last five alignment columns (a proxy for proteins with conserved C-terminal regions) were retained. The corresponding homologues from the reassigned genomes (predicted under the alternative genetic code, where UGA is not treated as a stop codon) were then added back to the respective alignments. After realignment, we examined proteins from the genomes with reassigned UGA for potential C-terminal extensions, which would indicate erroneous conceptual translation of a genuine UGA stop codon as tryptophan leading to an artificial extension of the coding sequence to a downstream UAA or UAG triplets.

The genes for suppressor tRNA_UCA_ were identified using tRNAscan-SE and ARAGORN v.1.2.41. The homologues of bacterial release factor 2 (RF2) were searched using the respective sequence of *E. coli* (NCBI accession XQK66079.1) as a query in BLASTp and tBLASTn searches against the predicted proteins and the genome assemblies of JAKLIH01 and their closest relatives, respectively, with an *e*-value threshold set to 1. The putative RF2 candidates were checked by the backward BLASTp searches against the *E. coli* predicted proteins, as well as by inspecting the annotations of the top five hits obtained by BLASTp search against the non-redundant protein sequence NCBI database [67].

### Transformation of *E. coli*

An enhanced green fluorescent (eGFP) reporter with a premature stop (UGA) codon in pBAD plasmid (Invitrogen/Thermo Fisher Scientific) was synthesized by Gene Universal. The wild type (WT) 5-bp AS *tRNA^Trp^_CCA_* gene was amplified from *E. coli* DH10B genomic DNA using primers (forward primer: 5′-GATCGCATGCAAAAAAGCCTGCTCGTTGAGCAGGCTTTTCGAATTTGAGGGGCGTAGTTCAATTGGT-3′, reverse: 5′-GATCGCTTAGCTGGCAGGGGCGGAGAGACTC-3 ′) with SphI and BlpI restriction sites, respectively. The polymerase chain reaction product was cloned into pBK plasmid (kindly provided by Nanxi Wang, Nanjing University of Chinese Medicine). The mutant 4-bp AS *tRNA^Trp^_CCA_* was generated by Q5-driven site-directed mutagenesis according to the manufacturer’s protocol (New England Biolabs) to introduce the C27A substitution (forward primer: 5′-TGGTAGAGCAACGGTCTCCAA-3′, reverse: 5′-ATTGAACTACGCCCCTTGG-3′).

### Western blotting

*Escherichia coli* cells were induced with 0.2% L-arabinose and incubated for 4 h at 37°C. Whole cell lysates were separated on Mini-Protein TGX Stain-free gels (Bio-Rad) and transferred to the PVDF membrane. The membrane was probed with mouse anti-GFP antibody (Thermo Fisher Scientific) followed by an anti-mouse IgG peroxidase-labelled rabbit antibody (Sigma–Aldrich/Merck) at 1:1000 and 1:2000 dilutions, respectively. The membrane was re-probed with an anti-*E. coli* OmpA rabbit antibody (Antibody Res) and a secondary anti-rabbit IgG peroxidase-labelled goat antibody (Sigma–Aldrich/Merck) at 1:50 000 and 1:2 000 dilutions, respectively.

Three independent biological replicates were quantified using Image Lab (Bio-Rad) and analysed for each of the following groups: eGFP-UGA (negative control), eGFP-UGA + C27A 4-bp AS *tRNA^Trp^_CCA_*, and eGFP-UGA + WT 5-bp *tRNA^Trp^_CCA_*. To assess the differences in full-length eGFP production among groups, we first performed a one-way ANOVA, which revealed a significant overall effect (F_2,6 _= 24.72, *P* = 0.0013). To identify differences among the conditions, we performed independent two-sample Welch’s *t*-test for pairwise comparisons. All statistical analyses were performed in GraphPad Prism v.7.03 (www.graphpad.com; last accessed: 3 September 2025) and visualized as mean ± standard deviation.

### Northern blotting

For northern blotting, 10 µg of total RNA was denatured in urea loading dye and separated on 8% polyacrylamide gels containing 8 M urea. Electrophoresis was carried out in 1× NNB buffer at 100 V for ~2 h. Gels were briefly stained with ethidium bromide to check RNA integrity, and RNA was transferred to Zeta-Probe nylon membranes in 0.5× NNB buffer. After UV crosslinking (120 000 µJ/cm^2^), the membranes were hybridized with an *E. coli* tRNA^Trp^ probe 5′-AGGGGCGGAGAGACTCGAAC-3′ and a 5S rRNA probe 5′-TGAGTTCGGCATGGGGTCAGG-3′ according to the standard protocol (Bio-Rad) and scanned on a Typhoon laser scanner (Cytiva).

### Molecular dynamics simulations of tRNA^Trp^_CCA_ variants

All-atom molecular dynamics (MD) simulations were performed for the WT tRNA^Trp^_CCA_ with 5-bp AS, mono-mutated variants unpinned at the top of AS denoted C27A, C27G, C27U, G43A, G43C, and G43U, and two 5-bp AS variants recognising UGA stop codon—Hirsh variant (G24A) and A9C. The initial tRNA tertiary structure was extracted in the A/T state from an *E. coli* X-ray crystallography ribosome–tRNA–EF-Tu model (PDB 4V5R [27]) including the non-canonical nucleotide modifications. The amino acid residue was removed and the nucleobase 9, 24, 27 or 43 was mutated by Open PyMOL v.2.5 using the Mutagenesis Wizard [68].

The isolated tRNA was immersed in a periodic rhombic dodecahedron box of water. The size of the box was chosen such that the distance between the tRNA and any box face was at least 1.2 nm. Sufficient K^+^ ions were added to neutralize the system. Additionally, excess concentration of 150 mM KCl and 20 mM MgCl_2_ was added to mimic the intracellular environment. Amber χ_OL3_ force field [69, 70] and Aduri’s force field [71] were used for canonical and non-canonical nucleotides, respectively. The simple point charge extended (SPC/E) water model [72] was used with Joung and Cheatham ion parameters [73].

All systems energy was minimized in 50 000 steps, gently heated to 300 K using 100 000 steps. The initial velocities were drawn randomly from the Maxwell–Boltzmann distribution at 10 K. The pressure was equilibrated to 1 bar using another 100 000 steps with 2 fs time step. The production simulations employed v-sites [74] that allowed for a time step of 4 fs. The temperature of 300 K was maintained by v-rescale thermostat [75], pressure of 1 bar by Parrinello–Rahman barostat [76]. All bonds were constrained to their equilibrium lengths by LINCS. The electrostatics was treated by Particle Mesh Ewald method [77] with a direct-space cut-off of 1.0 nm and van der Waals interactions were described by Lennard–Jones potential with a cut-off of 1.0 nm.

Twelve independent production trajectories, 250 ns-long each, were generated for each variant, differing in the initial set of velocities. All simulations were done in GROMACS 2021 [78]. The number of hydrogen bonds (H-bonds) was calculated by the *gmx hbond* tool with the default settings, namely with the donor–acceptor distance threshold of 0.35 nm and 30° as an angular threshold. The backbone root-mean-square deviations (RMSDs) were calculated using *gmx rms* tool after the least-square alignment of the backbone atoms to the initial tRNA conformation. The root-mean-square fluctuations (RMSFs) were calculated using *gmx rmsf* for all residues after the least-square alignment that involved non-hydrogen atoms excluding the stem residues 27–43. The RMSF profiles were averaged over the 12 independent trajectories. The relative ΔRMSF in % was obtained as 100⋅[RMSF(mutant) – RMSF(WT)]/RMSF(WT). The analysis was done for 150–250 ns trajectory fragments, excluding the initial portions used for system equilibration. The *P*-values were calculated from the Mann–Whitney U test for sets of 12 values, representing each of the trajectories.

## Results

### tRNAs with 4-bp anticodon stem are widespread in bacteria

**Figure 1.**
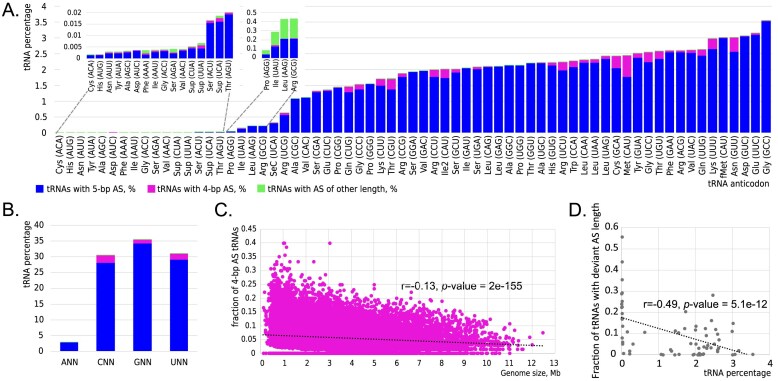
Distribution and properties of tRNA genes in bacteria. (**A**) Percentage of genes for tRNAs with the AS of various lengths in the total tRNA gene pool. (**B**) Percentage of genes for tRNAs with ANN, CNN, GNN, and UNN anticodons in the total tRNA gene pool. (**C**) Fraction of genes for 4-bp AS tRNAs (number of 4-bp AS tRNAs/total number of identified tRNAs) as a function of the genome size. The trend line demonstrates a negligibly weak negative correlation (*r*—Pearson correlation coefficient). (**D**) Fraction of tRNA genes with a deviant (i.e. different from 5 bp) AS (number of a certain tRNA species with a deviant AS/total number of identified tRNAs of the same species) as a function of a percentage of a given tRNA species out of all identified tRNAs.

We conducted a systematic survey of tRNAs with a 4-bp AS beyond tRNA^Trp^ in bacteria, initially disregarding whether unpinning occurred at the very top or very bottom part of the AS. An analysis of 42 109 genomes, encompassing over 150 classes of bacteria as defined by the GTDB [47], led to the identification of ~2.0 million tRNAs (Supplementary Table S1). Following the removal of putatively truncated tRNA genes, ~1.7 million tRNAs were analysed further. Bacterial genomes contain a median number of 39 tRNA genes (including copies of the same gene) with an average length of 77 nt (Supplementary Table S1). The most abundant tRNA genes in bacteria (here and below, abundance refers to the percentage of the respective genes out of all identified tRNAs) are those encoding *tRNA^Gly^_GCC_*, accounting for ~3.5% of the whole tRNA gene dataset, whereas *tRNA^His^_AUG_* and *tRNA^Cys^_ACA_* are the least abundant, each at only 0.002%, and, thus, absent in most bacterial genomes (Fig. 1A). Overall, the *tRNA_ANN_* species are among the least abundant, collectively making up just ~2.9% of the tRNA pool, whereas *tRNA_GNN_* comprise 35.4%, representing the most abundant tRNAs in the dataset (Fig. 1B).

We categorized bacterial tRNAs into three groups based on the length of the AS predicted using tRNAscan-SE: (i) 5-bp-long AS (1 596 864 tRNAs; 94.2%), (ii) 4-bp AS (95 748; 5.6%), and (iii) other AS lengths—2, 3, 6, and 7-bp (3136; 0.2%) (Supplementary Table S3). Only a negligible negative correlation (*r* = −0.13, *P*-value = 2*e−*155) was observed between the fraction of genes encoding 4-bp AS tRNAs (number of 4-bp AS tRNAs/total number of identified tRNAs) and the bacterial genome size (Fig. 1C). When each tRNA species was considered separately, there was a moderate negative correlation (*r* = −0.49, *P*-value = 5.1*e−*12) between the fraction of genes for tRNAs with a deviant AS length (number of a certain tRNA species with the AS different from five nucleotides/total number of identified tRNAs of the same species) and a percentage of a given tRNA gene in a total tRNA gene pool (Fig. 1D). Yet, only a weak negative correlation (*r* = −0.18, *P*-value = 1.4*e−*8) was observed when exclusively 4-bp AS tRNAs were considered. All tRNA types exhibit variability in the length of their AS, with the highest fraction of 4-bp AS tRNAs observed for the elongator tRNA^Met^_CAU_ (27.3%), tRNA^Sup^_UUA_ (20.5%), and tRNA^Thr^_CGU_ (18.5%) (Fig. 1A and Supplementary Table S3).

The genome analysis indicates that unpinning of one of the terminal base pairs in the AS is a widespread, yet previously largely underappreciated deviation from the canonical secondary tRNA structure in bacteria. Although all tRNA species exhibit variations in the number of base pairs within their AS, the prevalence of these deviations is clearly non-random, being much higher in low-abundant tRNA species.

### Distribution of 4-bp AS tRNA^Trp^_CCA_ across bacteria

The 4-bp AS tRNA^Trp^_CCA_ (with AS unpinned at the top) was experimentally shown to decode the non-cognate UGA in eukaryotic translation systems [35, 36]. The 4-bp AS tRNA^Trp^_CCA_ has also been recently proposed, although without experimental proof, to perform the same function in an endosymbiotic bacterium, dubbed XS4, possibly inhabiting a dinoflagellate [45]. Therefore, we first focused on the distribution of the respective tRNA form in bacteria.

**Figure 2.**
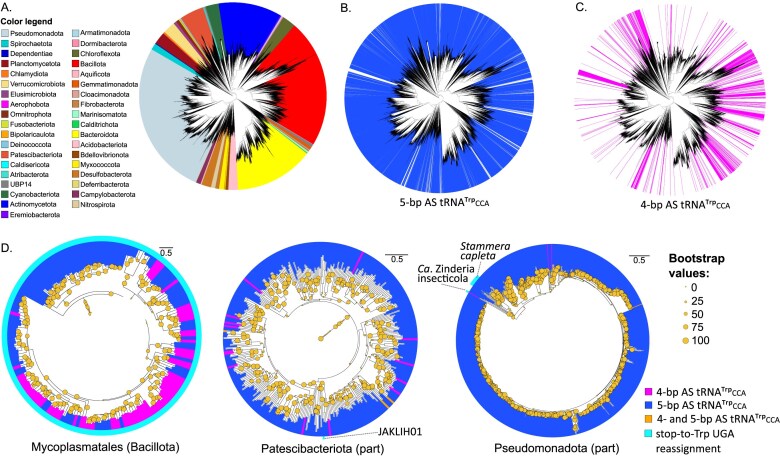
Distribution of 4- and 5-bp tRNA^Trp^_CCA_ variants across bacterial phylogeny. (**A**) Visualization of the bacterial phylogenomic tree from the GTDB depicting evolutionary relationships among bacteria. Only the 37 largest phyla, based on the number of species in the dataset, are shown. (**B**) The distribution of 5-bp tRNA^Trp^_CCA_ genes across bacteria. (**C**) The distribution of 4-bp AS (unpinned at the top or bottom) tRNA^Trp^_CCA_ genes across bacteria. (**D**) The distribution of 4-bp tRNA^Trp^_CCA_ in some of the lineages where stop-to-tryptophan UGA reassignment has been documented, including the order Mycoplasmatales (phylum Bacillota) and particular lineages of the phyla Patescibacteriota and Pseudomonadota. For the latter two phyla, a broader part of the phylogenetic tree that contains the species with stop-to-tryptophan UGA reassignment and their relatives with the standard code is shown. We note that the Pseudomonadota tree does not display some of the cases with the same reassignment, including *Ca*. Hodgkinia cicadicola and *Ca*. Nasuia deltocephalinicola, as they are not included in the GTDB and hence are missing from the reference tree used for the display. For the same reason, we do not show data for the known Verrucomicrobiota lineage (e.g. *Ca*. Pinguicoccus supinus) with stop-to-tryptophan UGA reassignment. Species possessing 4- and 5-bp tRNA^Trp^_CCA_ are highlighted in magenta and blue, respectively. Species containing both 4- and 5-bp tRNA^Trp^_CCA_ are not visible due to their low number and compactness of the trees. Only the genomes with at least one identified tRNA^Trp^_CCA_ gene are shown. Bootstrap supports are indicated by the size of the circles. Horizontal line represents 0.5 substitutions per site. Species demonstrating stop-to-tryptophan UGA reassignment are indicated with cyan.

In the analysed dataset of 42 109 bacterial genomes, *tRNA^Trp^_CCA_* comprise 38 615 (2.3%) of all identified tRNA genes, with 3438 (8.9%) of them possessing a 4-bp AS, out of which 3171 have AS unpinned at the top (Supplementary Tables S1 and S3). In contrast to the 5-bp AS variant, the distribution of 4-bp AS *tRNA^Trp^_CCA_* in bacteria is patchy (Fig. 2A–C), being more prominent in Hydrogenedentota (where exclusively 4-bp AS variant was identified), Campylobacterota, Babelota, Myxococcota, and Cloacimonadota (Supplementary Fig. S1). Overall, out of 142 bacterial phyla in the analysed dataset, 34 (23.9%) contained at least one representative with 4-bp AS *tRNA^Trp^_CCA_* (Supplementary Table S1). We zoomed onto several groups, where the stop-to-tryptophan UGA reassignment has been reported before or has been newly discovered in our analyses (Fig. 2D). The latter category corresponds to a family-level lineage of the phylum Patescibacteriota referred to as JAKLIH01 in the GTDB release 10-RS226 (see below). Among bacteria with stop-to-tryptophan UGA reassignment, there are species, which contain either 5-bp AS *tRNA^Trp^_CCA_* (some Mycoplasmatales, JAKLIH01, *Stammera capleta*) or its 4-bp variant (some Mycoplasmatales, *Ca*. Zinderia insecticola, and the uncultivated bacterium XS4 from the family Fastidiosibacteraceae) (Fig. 2D and Supplementary Table S1) [45].

Among bacterial genomes with the standard genetic code (i.e. not showing signs of any type of codon reassignment), 2677 (~6.5%) encode exclusively *tRNA^Trp^_CCA_* with 4-bp AS, indicating that this variant is sufficient for decoding standard UGG tryptophan codons, as has been demonstrated in the eukaryotes examined [35, 36]. In genomes that exclusively encode 4-bp AS *tRNA^Trp^_CCA_*, the proportion of UGA codons is highly variable, ranging from as low as 1% to as high as 88% of all identified stop codons. At the taxonomic level of an order, analysis of bacterial groups with at least 10 genomes encoding only 4-bp AS *tRNA^Trp^_CCA_* showed that in 13 of the 19 orders, the proportion of UGA stop codons was significantly lower compared to that in the genomes of bacteria from the same order possessing only the 5-bp AS *tRNA^Trp^_CCA_* gene (Supplementary Table S4). Thus, the presence of 4-bp AS *tRNA^Trp^_CCA_* is associated with a decreased UGA usage.

We performed intrageneric synteny analysis for 10 bacterial genera that included genomes encoding either 4- and 5-bp AS *tRNA^Trp^_CCA_*. Among these, we found three cases in which the 4- and 5-bp AS *tRNA^Trp^_CCA_* genes were located within a syntenic region, that is, appeared to be orthologous. Thus, at least in some cases, the conversion between 4- and 5-bp seems to occur via a point mutation in the AS (Supplementary Fig. S2).

The 4- and 5-bp AS *tRNA^Trp^_CCA_* forms co-occur only in a small subset of bacteria with the standard genetic code (296 genomes representing ~0.7% of the analysed species with the standard code) (Supplementary Table S1). To minimize potential biases associated with species-specific evolutionary rates, we compared pairwise sequence identities between 4- and 5-bp AS *tRNA^Trp^_CCA_* in bacteria encoding both variants (Supplementary Fig. S3). The median pairwise sequence identities were 63.9% and 64.4% for 4- and 5-bp AS *tRNA^Trp^_CCA_*, respectively, with the difference being statistically significant (*t*-test *P*-value = 7.2*e−*40), but negligibly small (Cohen’s *d* = 0.09). Thus, purifying selection acts to maintain sequence conservation in both tRNA variants. The analysis of RNA-seq data from several bacterial genomes confirmed that both 4-bp and 5-bp AS *tRNA^Trp^_CCA_* genes were expressed, with the 4-bp AS variant showing lower expression level (Supplementary Fig. S4).

**Figure 3.**
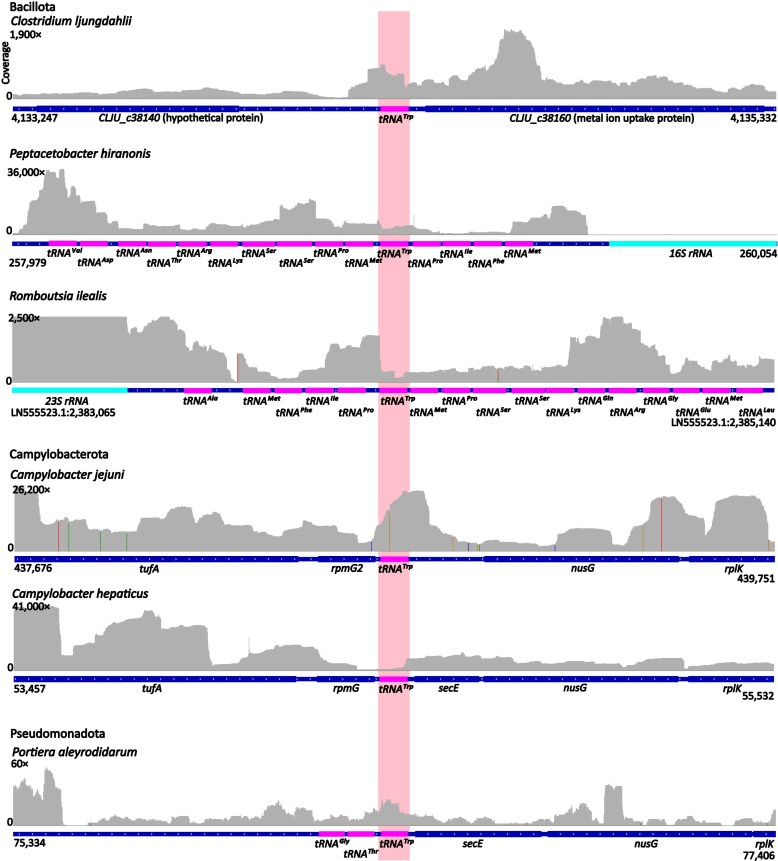
*Blastocrithidia*-like (4-bp AS) *tRNA^Trp^_CCA_* are expressed in bacteria. A snapshot of RNA-seq data mapping onto the genome assembly at the *tRNA^Trp^_CCA_* locus (highlighted in light pink) and 1000 nucleotides up- and downstream in various bacteria. Coordinates of the displayed region in the genome assembly are indicated (scaffold names are shown only when the assembly contains multiple scaffolds). Protein-coding, tRNA, and rRNA genes are depicted by blue, magenta, and turquoise bars, respectively. The upper track shows RNA-seq read coverage (with the maximum value indicated on *Y* axis). Nucleotide positions in the reads different from the reference are highlighted in the coverage track (supported by the read fraction ≥0.3): A, green; T, red; G, blue; C, orange. Transcriptomic data from public databases were used to generate coverage plots (see the ‘Materials and methods’ section for details). Products of protein-coding genes: *nusG*, N-utilization substance G; *rplK*, large ribosomal subunit protein uL11; *rpmG2*, large ribosomal subunit protein bL33B; *secE*, secretion protein E; *tufA*, elongation factor Tu 1.

In addition to 4-bp AS tRNA^Trp^_CCA_ unpinned at the very top of the AS, we also identified 4-bp AS tRNA^Trp^_CCA_ unpinned at the very bottom, i.e. lengthening the AL by 2 nt (position 30A·38U in *B. nonstop* tRNA^Trp^_CCA_), which were not subject of this study. Moreover, in any 4-bp AS tRNA^Trp^_CCA_ that harbour additional mutations (such as A9C and G24A), which were shown to facilitate UGA readthrough [33, 34], the contribution of 4-bp AS to the overall effect is unknown. Therefore, from here on, we refer to tRNA^Trp^_CCA_ with 4-bp AS and 7-nt AL (i.e. unpinned at the top) and lacking A9C and G24A mutations as the ‘*Blastocrithidia*-like tRNA^Trp^_CCA_’. We categorized 3438 4-bp AS tRNA^Trp^_CCA_ based on the variation in sequence and structure, excluded 267 tRNAs with AS unpinned at the bottom and 2 414 tRNAs carrying A9C and/or G24A, and focused on 757 genes encoding *Blastocrithidia*-like tRNA^Trp^_CCA_ representing 22.0% of all 4-bp AS tRNA^Trp^_CCA_ and 2.0% of all identified tRNA^Trp^_CCA_ (Supplementary Table S5). Only in 62 genomes, all with the standard genetic code, *Blastocrithidia*-like tRNA^Trp^_CCA_ co-occurred with the 5-bp AS variant, yet no RNA-seq data were available for these genomes. However, RNA-seq data are available for several genomes that do not encode the 5-bp AS tRNA^Trp^_CCA_ gene along with its 4-bp AS variant (Fig. 3). In these genomes, *Blastocrithidia*-like *tRNA^Trp^_CCA_* genes are covered by RNA-seq reads suggesting that they are expressed (Fig. 3). Moreover, analysis of RNA-seq read mapping did not reveal nucleotide substitutions resulting from the RNA editing events in the AS that would restore base pairing between the top unpinned nucleotides or change the anticodon to alter the codon specificity of the tRNA (Fig. 3).

Overall, findings that some genomes encode exclusively 4-bp AS tRNA^Trp^_CCA_ and that its sequence conservation level is comparable to that of the 5-bp variant, combined with RNA-seq evidence of their expression, suggest that 4-bp AS tRNA^Trp^_CCA_ are functional, with the implication that they are capable of decoding canonical tryptophan UGG codon as tryptophan in bacteria.

### The distribution of 4-bp AS tRNA^Trp^_CCA_ across bacteria with alternative genetic codes

Given that the functionality of *Blastocrithidia-*like tRNA^Trp^_CCA_ was documented in eukaryotes with the stop-to-tryptophan UGA reassignment, we analysed the presence of such tRNAs in bacteria with alternative genetic codes.

The genetic code inference in the bacterial dataset led to the identification of a new independent case of the stop-to-tryptophan UGA reassignment in the phylum Patescibacteriota, specifically in a family-level lineage referred to as JAKLIH01 and classified in the order UBA6257 and the class Minisyncoccia in the GTDB (release 10-RS226), which was not included in the previous large-scale analysis of codon reassignments in bacteria [44]. It exhibits a strong signal for UGA being decoded as tryptophan when analysed by Codetta (Supplementary Table S2). Analysis of four representatives of this family confirmed that they form a clade in a phylogenetic tree, suggesting that the reassignment in this group was a result of a single evolutionary event (Supplementary Fig. S5). The identification of a *tRNA_UCA_* gene in two out of the four genomes, the absence of RF2 genes, and the absence of UGA stop codons at the ends of coding sequences further support the presence of this reassignment (Supplementary Table S2).

**Figure 4.**
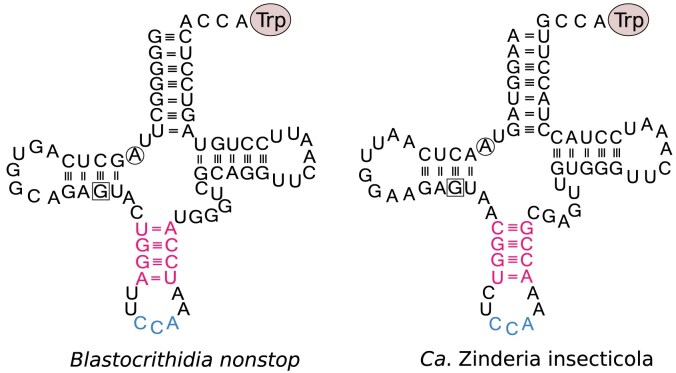
Secondary structure of 4-bp AS tRNA^Trp^_CCA_ from the trypanosomatid *Blastocrithidia nonstop* and the bacterium *Candidatus* Zinderia insecticola. In both cases, a 4-bp AS *tRNA^Trp^_CCA_* is present in the context of stop-to-tryptophan UGA reassignment and the absence of the ‘suppressor’ *tRNA*_*UCA*_.

Overall, among the bacterial genomes analysed, 201 (0.5%) were predicted to encompass stop-to-tryptophan UGA reassignment (Supplementary Table S1). These genomes belong to four phyla and jointly include around 30 bacterial genera (Supplementary Table S1). Of these genomes, 70 (34.8%) encode tRNA^Trp^_CCA_ with a 4-bp AS; in all cases, as the only tRNA^Trp^_CCA_ variant. Among these, 11 were *Blastocrithidia-*like tRNA^Trp^_CCA_, and the remaining ones had deviations from the structure defined above. In 65 of the 70 genomes, 4-bp AS tRNA^Trp^_CCA_ co-occurs with a tRNA^Trp^_UCA_ fully cognate to the UGA stop codon (Supplementary Table S1). In the five remaining cases, namely *Candidatus* Zinderia insecticola and four representatives of the order Mycoplasmatales (Supplementary Table S1), we did not identify a *tRNA^Trp^_UCA_* gene. However, only the genome of the endosymbiotic bacterium *Ca*. Z. insecticola was assembled into a single circular sequence and, thus, is likely complete. It suggests that, at least in this species, the *Blastocrithidia-*like tRNA^Trp^_CCA_ mediates decoding of the in-frame UGA codons as tryptophan in the absence of a fully cognate suppressor tRNA (Fig. 4). The representatives of Mycoplasmatales encoding *Blastocrithidia-*like tRNA^Trp^_CCA_ have fragmented genome assemblies (consisting of up to 73 contigs) and the absence of a *tRNA^Trp^_UCA_* gene might be an artefact of incomplete genome assembly. Nonetheless, these species with likely incomplete genome assemblies do not form a clade in the phylogenetic tree, which suggests that if the 4-bp AS tRNA^Trp^_CCA_-dependent mechanism for decoding UGA as tryptophan was functional, it must have evolved independently at least four times in Mycoplasmatales. Given that the vast majority of Mycoplasmatales encode a tRNA^Trp^_UCA_, it is parsimonious to assume that this particular tRNA form likely represents the shared adaptation underlying stop-to-tryptophan UGA reassignment in this order.

Collectively, these findings, along with the recently published data on the putative dinoflagellate endosymbiont XS4 from the family Fastidiosibacteraceae [45], imply that the stop-to-tryptophan UGA reassignment mechanism relying on a *Blastocrithidia-*like tRNA^Trp^_CCA_ is not exclusive to eukaryotes but also functions in some bacteria. However, experimental validation is needed to support this observation, as well as to determine whether 4-bp AS tRNA^Trp^_CCA_ decodes UGA as tryptophan in bacteria where it coexists with tRNA^Trp^_UCA_.

### 4-bp AS tRNA^Trp^_CCA_ enables UGA readthrough in *E. coli*

**Figure 5.**
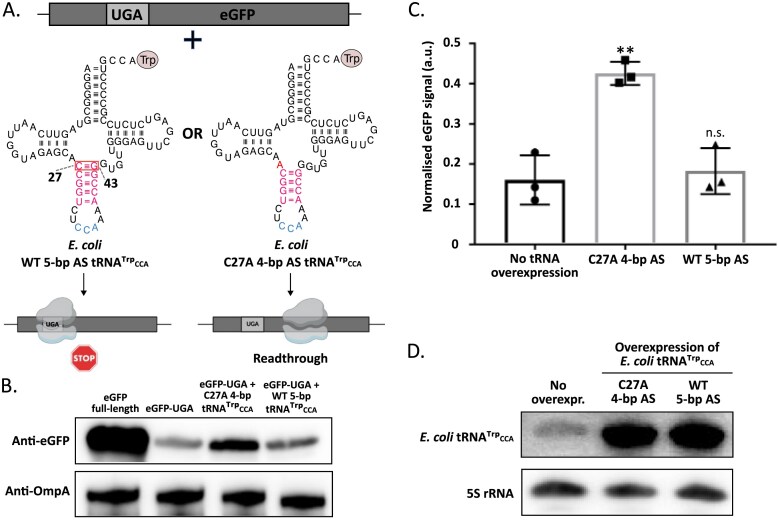
A confirmation of the ability of a 4-bp AS tRNA^Trp^_CCA_ to enable translational UGA readthrough in *E. coli*. (**A**) Truncated eGFP with an in-frame UGA stop codon expressed in *E. coli* from pBAD plasmid in the presence of arabinose. The eGFP was co-expressed with either the *E. coli* WT 5-bp AS tRNA^Trp^_CCA_ or the *E. coli* C27A 4-bp AS tRNA^Trp^_CCA_ in pBK to assess the ability of the different versions of the tRNAs to mediate translational readthrough of the UGA codon. The outcome of the experiment predicted by the extended superwobble hypothesis is shown for the two tRNA variants. (**B**) Western blot analysis showing translational readthrough of the in-frame UGA codon. An anti-eGFP antibody was used along with an anti-OmpA antibody as a loading control. In the case of the anti-eGFP antibody, the second lane reflects the UGA readthrough at the background of the endogenous tRNA^Trp^_CCA_ level, and the variant lanes show increased signal upon plasmid-driven overexpression of 4- and 5-bp AS tRNA^Trp^_CCA_. (**C**) Quantification of the UGA readthrough in the eGFP construct based on three replicates of the western blot analysis (mean ± the standard deviation; the individual values are depicted as black circles, squares, and triangles). Differences were assessed with two-samples Welch’s *t*-test for pairwise comparisons (eGFP-UGA + C27A 4-bp *tRNA^Trp^_CCA_*versus negative control: *t* = 6.76, df = 2.86, *P* = 0.0077; eGFP-UGA + C27A 4-bp *tRNA^Trp^_CCA_*versus eGFP-UGA + WT 5-bp tRNA^Trp^_CCA_: *t* = 6.57, df = 2.97, *P* = 0.0074). **P < .01. (**D**) Northern blot analysis showing the overexpression (compared to the endogenous level of 5-bp tRNA^Trp^_CCA_ in the first lane) of the two versions of the *E. coli* tRNA^Trp^_CCA_ upon transformation with pBK plasmid. The expression is detected using oligonucleotide probes specific to *E. coli* tRNA^Trp^_CCA_ and the 5S rRNA. Abbreviations: AS, anticodon stem; n. s., not significant; WT, wild type.

Previously, in a bacterial system, it has been shown that the naturally unpinned top base pair of the AS of tRNA^Thr^ (by the unusual G27-A43 mismatch), when combined with the anticodon substitution from CGU to CUA, mediates the UAG readthrough more efficiently than its engineered 5-bp AS variant [79]. Similarly, mutations unpinning the top AS base pair of *E. coli* tRNA^Trp^ at the same time bearing the anticodon mutated from natural CCA to CUA could efficiently read through the UAA stop codons [43]. The ability of the *E. coli Blastocrithidia-*like tRNA^Trp^_CCA_ (i.e. with the preserved CCA anticodon) to read through UGA has been tested in an *in vitro* system. Introducing C27A mutation alone (unpinning the top AS base pair) did not lead to UGA readthrough, however, C27A was essential for the function of the C27A, G24A, G59A triple mutant [80]. To test the impact of the single C27A substitution *in vivo*, we expressed an eGFP reporter with a premature UGA stop codon introduced at a randomly selected amino acid position 29 in *E. coli* in the presence of selected tRNA^Trp^_CCA_ variants (Fig. 5A). Only upon efficient UGA readthrough, the full-length eGFP can be made.

While overexpression of the WT 5-bp AS tRNA^Trp^_CCA_ did not increase the full-length eGFP expression above the ‘no overexpression’ control (Fig. 5B and C), its unpinned 4-bp variant (the C27A substitution) substantially (~2.5-fold) boosted the mutant eGFP expression. The overexpression of both tRNA^Trp^_CCA_ variants was confirmed by northern blot analysis (Fig. 5D). These findings clearly demonstrate that 4-bp AS tRNA^Trp^_CCA_ enables higher UGA readthrough compared to the 5-bp AS variant in a prototypical bacterial system, strongly suggesting that the molecular mechanism underlying the action of this tRNA variant, naturally occurring in several species with the stop-to-tryptophan UGA reassignment [35, 39–41, 45], is conserved in at least two domains of life. However, because the overexpression of 4-bp AS tRNA^Trp^_CCA_ in our reporter assay likely results in levels that exceed its endogenous abundance in bacteria that carry and express this form of tRNA from their genomes, the functional significance of its intrinsic capacity to promote more efficient stop codon readthrough remains to be assessed at native, physiologically relevant expression levels in these specific lineages.

### Tertiary structure of *Blastocrithidia*-like 4-bp AS tRNA^Trp^_CCA_ variants

To gain insight into the mechanism of UGA readthrough in *E. coli*, we performed atomistic MD simulations of six variants unpinning the top bp of tRNA^Trp^_CCA_, including the 4-bp *Blastocrithidia*-like C27A:G43 variant, and compared them with the WT 5-bp variant that disfavours stop-to-tryptophan reassignment (Fig. 5A), and A9C and Hirsh G24A variants that enable UGA readthrough. All tRNAs were analysed in aqueous solution, i.e. outside the ribosome. Note that 2 out of 6 of our 4-bp variants (G43U and C27A) were shown experimentally to mediate UGA readthrough [43, 81].

Initial visual inspection of MD trajectories revealed that the overall tertiary structure of all tRNA variants was maintained. The number of H-bonds of the mutated pair is reduced relative to the WT, but remains non-zero in many cases (Supplementary Fig. S6). Consequently, unpinning a pair in the 2D secondary-structure diagram (Supplementary Fig. S6A) does not mean that H-bonds are absent in the 3D structure (Supplementary Fig. S6B). This might be attributed to the stabilization of the tertiary tRNA structure by base stacking of purine residues and non-canonical H-bonding patterns of the unpinned pair.

**Figure 6.**
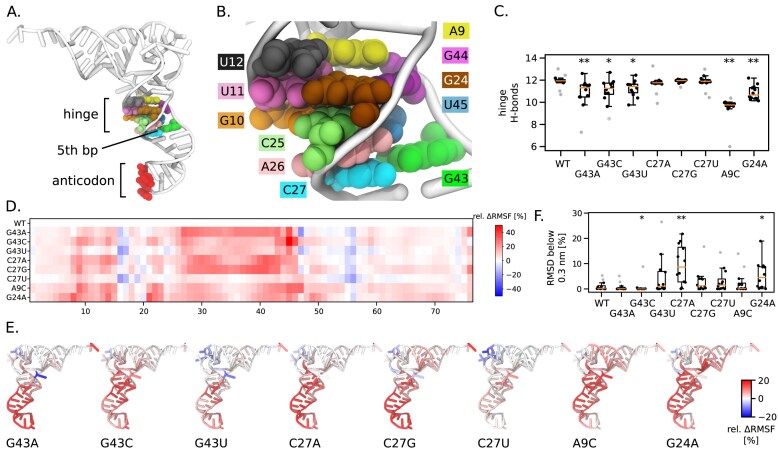
Atomistic MD simulations of several 4-bp *Blastocrithidia*-like tRNA^Trp^_CCA_ variants compared to the 5-bp AS WT, A9C and Hirsh G24A variants. (**A**). Tertiary structure of the tRNA. (**B**) Context of the unpinned residues C27:G43 at the top of the AS, and the tRNA hinge. (**C**) Number of hydrogen bonds (H-bonds) within the hinge inferred by MD simulations. The boxes indicate the interquartile range, the red line denotes the median, whiskers extend to the most extreme data points within 1.5× the interquartile range, and grey circles represent outliers. Statistics were calculated from 12 values, each obtained as the average over one of the 12 independent trajectories. **P < .01, *P < .05. (**D**) The residue-wise ΔRMSF relative to the WT. The colour scale goes from blue (more rigid than WT) through white to red (more flexible than WT). (**E**) A projection of the relative ΔRMSF onto the tertiary tRNA structure. The initial WT tRNA conformation was used for all projections. (**F**) A percentage of simulation time, where the tRNA backbone adopted the RMSD lower than 0.3 nm. High percentage means that the tRNA tends to stay in the A/T conformation, which is required for efficient stop codon readthrough.

Nevertheless, we identified perturbations caused by the unpinning. Notably, the top AS base pair is adjacent to a well-known hinge region at the interface of D arm, AS, and variable arm (Fig. 6A) that has previously been linked to the effects of the mutations A9C and G24A (Fig. 6B) [27]. To probe this connection, we analysed H-bonding within the tRNA hinge defined here by residues 9–12, 23–26, 44, and 45, using the average number of H-bonds as a proxy for thermodynamic stability. Mutations at position G43 increased the variance in the mean H-bond count similar to the Hirsh G24A (Fig. 6C). The loss of two H-bonds was observed for the A9C variant. This destabilization of the hinge region of G43 mutants was accompanied by an increase of the motion of the AS relative to the rest of the tRNA, as reflected by the changes in RMSF (Fig. 6D and E) that stands for a flexibility measure (see the ‘Materials and methods’ section). The increase in the flexibility of the A9C variant was previously linked to the adoption of the A/T tRNA state in the ribosomal ternary complex, thereby promoting GTP hydrolysis by EF-Tu [27]. We observe the same increase in AS flexibility of all G43 mutants and, therefore, conclude that G43 mutations may be prone to stop-codon readthrough via a mechanism similar to the A9C mutation.

Conversely, the C27 mutations did not alter the H-bonding count of the hinge region (Fig. 6C). Yet, at least the C27A variant supports stop-to-tryptophan reassignment. Inspired by the mechanism proposed for the Hirsh G24A mutation [27], we next focused on the tRNA structure. We calculated backbone RMSD values after superimposing the trajectories onto the initial A/T conformation. This metric reflects the global arrangement of the tRNA during the simulations, and consequently, low RMSD values correspond to stabilization in the A/T conformation required for successful stop codon reassignment. In the aqueous environment, the tRNA expectedly departed from the A/T conformation, but we noticed that the extent of this departure differed between variants. Strikingly, the three variants that sampled the A/T conformation most extensively were C27A, G24A, and G43U (Fig. 6F)—precisely those experimentally demonstrated to mediate UGA readthrough [43, 81]. By contrast, the G43C variant showed the largest departure from the A/T state (i.e. the lowest RMSD), likely precluding efficient readthrough despite its high AS flexibility.

To place our findings in a broader context, we analysed a dataset of 757 genes encoding *Blastocrithidia*-like tRNA^Trp^_CCA_ as well as the entire dataset of the tRNA^Trp^_CCA_ with the AS unpinned at the top (Supplementary Tables S5 and S6). The G43A and G43U variants (featuring C:A and C:U unpinned pairs), which our simulations identified as highly flexible, were present in 35.0 and 18.6% of sequences, respectively, and together with G:A unpinned pair represented three most populated variants. Moreover, the analysis revealed a marked ~4-fold increase in the frequency of the C:A unpinned pair in ‘*Blastocrithidia*-like’ tRNA^Trp^_CCA_ (35%) compared to the entire set of 4-bp AS tRNA^Trp^_CCA_ (8.7%) with the AS unpinned at the top (Supplementary Table S6). In contrast, the G43C variant was found in only a single gene (0.1%) among the ‘*Blastocrithidia*-like’ sequences. In simulations of this variant, the hinge exhibited pronounced instability: within 250 ns, U45 detached completely from the hinge, affecting also m^7^G46 and U47 making this region the major difference between G43C and the two readthrough-competent variants G43A and G43U (Fig. 6D and E).

Mutations at C27 were rare in both the ‘*Blastocrithidia*-like’ and the entire dataset of the 4-bp AS tRNA^Trp^_CCA_ (with the frequency of such variants not exceeding 1.1%). C27U was absent in the datasets, which is in line with the MD results in which this variant was the only one that showed no increase in flexibility upon mutation (Fig. 6D and E). These results suggest that the G24A-like mechanism, which relies on pre-stabilization of the A/T tRNA conformation, is evolutionarily more difficult to achieve through mutations near the tRNA hinge.

## Discussion

Being essential for polypeptide chain formation, tRNA molecules retain highly conserved secondary and tertiary structures, with alterations occurring mostly in the variable arm or, less frequently, in the D- or T-stem/loop architecture [82]. The anticodon arm, known for its high conservation level, typically features a 5-bp AS and a 7-nt AL [6]. Recently, however, we reported the presence of a functional tRNA^Trp^ species with a 4-bp AS in the trypanosomatid *B. nonstop* and the ciliate *C. magnum*, where this tRNA variant enables UGA stop-to-sense reassignment [35]. So far, the codon recognition specificity of tRNAs was almost exclusively predicted on the basis of their anticodon [83]. However, this traditional rationale would not work for predicting the codon specificity of 4-bp AS tRNA^Trp^_CCA_ [46], and potentially, the other 4-bp AS tRNAs with this (unpinned at the very top) or similar (unpinned at the very bottom) structural features. Here, we investigated the potential decoding capacity of 4-bp AS tRNAs in bacteria.

Although far less abundant than the canonical 5-bp AS tRNAs, 4-bp AS tRNAs (unpinned either at the top or at the bottom of the AS) are surprisingly common in bacteria. We identified this alteration in all tRNA species, with at least one 4-bp tRNA found in 85.6% of the analysed genomes. Thus, unpinning of one of the AS base pairs is a widespread, previously almost entirely overlooked deviation from the canonical tRNA structure in bacteria. The substantial prevalence of 4-bp AS tRNAs strongly suggests their functionality. Indeed, MD simulations revealed that (at least in the case of tRNA^Trp^_CCA_) while unpinning the top base pair may reduce the number of H-bonds in the AS, the overall tRNA tertiary structure seems to be maintained by stacking interactions of surrounding bases and/or formation of the non-canonical H-bonding patterns in the unpinned base pair.

Considering that several unicellular eukaryotes carry a 4-bp AS tRNA^Trp^_CCA_ to mediate translational UGA readthrough [35, 36, 41], here we specifically focused on the analysis of bacterial 4-bp AS tRNA^Trp^_CCA_ to identify similar cases. This variant constitutes 8.9% of all identified tRNA^Trp^_CCA_ in bacteria, and its substantial subset (22%) is structurally closely similar to the *B. nonstop* tRNA^Trp^_CCA_. In bacteria, 4- and 5-bp AS tRNA^Trp^_CCA_ variants co-occur in the same genome extremely rarely, and, because the conservation levels of 4- and 5-bp AS *tRNA^Trp^_CCA_* in the genomes encoding both variants are comparable and both are transcribed, they appear to be subject to similar selection pressures.

Our analyses of 4-bp AS tRNA^Trp^_CCA_ show that certain nucleotide combinations are more frequent in the unpinned position at the top of the AS, suggesting some sequence constraints at this site. Although the three-nucleotide anticodon of a tRNA is the primary determinant for base pairing with an mRNA codon, the ability of a tRNA to recognize and translate that codon is not dictated by the anticodon alone. In particular, it was previously proposed that residues on the 3′ side of the anticodon (positions 37 and 38), as well as base pairs within the AS evolved in correlation with the identity of the cardinal anticodon nucleotide at position 36. Indeed, experiments aimed at testing this hypothesis demonstrated that stop codon suppression efficiency can be improved by modifying the AS–loop sequence to better match the identity of the cardinal nucleotide according to these rules [84–86]. In addition, *in vitro* biochemical analyses of bacterial tRNA binding to the ribosomal A site showed that the identity of 32 and 38 bases (pairing only in some tRNAs, and constituting an open AL in others) correlates with anticodon identity, similarly fine-tuning the interaction of each tRNA with the decoding centre in the ribosome [87]. Disruption of this correlation impaired ribosomal acceptance of cognate tRNAs and compromized translational fidelity, further highlighting the importance of coordinated alignment between the anticodon and the surrounding free bases and structural elements such as the AS. Structural studies of these tRNA variants bound to the ribosome reinforced this concept even further [88]. In line with these reports, our observations on the frequency of the nucleotide combinations at the top of the AS combined with the MD simulations support a functional link between the identity of the unpinned base pair, the architecture of the AS–loop, and the propensity of tRNAs to promote stop or near-cognate codon readthrough. However, the situation seems to be highly complex, and additional structural studies will be required to fully uncover the molecular basis for the increased UGA codon readthrough by the 4-bp AS tRNA^Trp^_CCA_ variants.

The distribution of 4-bp AS *tRNA^Trp^_CCA_* genes in bacteria implies that the ancestral 5-bp AS *tRNA^Trp^_CCA_* morphed into the 4-bp AS variant by means of point mutations, which is supported by the observation that the two variants are found within syntenic genomic regions. Moreover, in some bacterial lineages, the transition between 4- and 5-bp AS *tRNA^Trp^_CCA_* occurred notably more frequently than in others. Multiple cases of independent emergence of the 4-bp AS *tRNA^Trp^_CCA_* variant in lineages with the stop-to-tryptophan UGA reassignment (exemplified by Mycoplasmatales) suggest that it confers increased fitness, possibly, through augmenting the UGA to tryptophan recoding via the cognate tRNA^Trp^_UCA_. An alternative explanation is that bacteria in which UGA encodes tryptophan tolerate the decoding of UGA by the 4-bp AS tRNA^Trp^_CCA_ more readily than bacteria that use UGA as a stop codon, because in the latter the 4-bp AS tRNA^Trp^_CCA_ may interfere with proper translation termination by competing with RF2.

In bacteria without the stop-to-tryptophan UGA reassignment, possessing exclusively 4-bp AS tRNA^Trp^_CCA_ would be deleterious unless RF2 outcompetes this tRNA at the UGA codons. We identified a statistically significant reduction in the UGA stop codon usage in genomes encoding only 4-bp AS tRNA^Trp^_CCA_ compared to their relatives possessing solely the 5-bp AS variant for 13 of the 19 analysed bacterial orders. Nevertheless, the fact that all 4-bp AS *tRNA^Trp^_CCA_*-containing genomes still use UGA as a stop codon suggests that their RF2 is a sufficiently strong competitor of 4-bp AS tRNA^Trp^_CCA_ for UGA recognition. Alternatively, some unknown modifications to this tRNA might prevent its interaction with UGA.

It has been previously demonstrated that unpinning the top base pair of the AS can alter the codon–anticodon interaction in bacteria [43, 81]. Here, we showed experimentally that 4-bp AS tRNA^Trp^_CCA_ mediates higher UGA readthrough in *E. coli*, when compared to WT tRNA^Trp^_CCA,_ which further underscores the capacity of 4-bp AS tRNAs to superwobble in a bacterial system. Notably, a previous *in vitro* selection of *E. coli* tRNA^Trp^_CCA_ using UGA-programmed 70S ribosomes showed no effect of C27A alone (unpinning the top base pair) on UGA decoding [80]. These different outcomes likely reflect differences in an *in vitro* versus *in vivo* experimental setups. Consistent with the functional impact, our MD analysis indicates that C27A stabilizes the A/T conformation of the tRNA required for efficient GTP hydrolysis in the ribosome–EF-Tu–tRNA ternary complex.

Our findings suggest that in bacteria, as in eukaryotes, 4-bp AS tRNA^Trp^_CCA_ represents a dedicated, even if relatively rare mechanism for stop-to-tryptophan UGA reassignment. Most bacteria with a genome-wide UGA reassignment use an alternative strategy, in which the so-called suppressor tRNAs fully cognate to UGA decode this codon [44]. Why do eukaryotes and some bacteria favour the 4-bp AS tRNA-mediated mechanism of UGA decoding over the ‘suppressor’ tRNA-mediated one, remains an open question. ‘Suppressor’ tRNAs are also present in some bacteria with the standard genetic code (in 0.3% of the genomes analysed here), where they decode in-frame UGA (and other stop codons) that arise through nonsense mutations [89].

Our present results suggest an extension of the superwobble hypothesis. The minimal set of 32 tRNAs was initially proposed to be sufficient for decoding all sense codons of the genetic code [12]. Some codons are read by cognate tRNAs carrying anticodons that are exactly complementary to the codon, whereas others follow the wobble rules [12]. Under these rules, certain non-canonical base pairs, such as G:U, can occur in the wobble position, whereas other pairs, such as C:A, are less favourable. The superwobble hypothesis introduced later stated that tRNAs with U at the wobble position can decode all four codons in their respective four-codon families [11, 90]. Our results, reported here and previously [35], expand the superwobble hypothesis by including the C:A base pair, which, in particular, allows 4-bp AS tRNA^Trp^_CCA_ to decode the UGA codon. The extended superwobble hypothesis thus postulates that, regardless of their anticodon, tRNAs with a 4-bp AS and a C at the wobble position have an expanded decoding range, recognising both G- and A-ending codons. This extension of the superwobble hypothesis assumes that the possibility of C:A pairing at the 3rd codon position is valid for other tRNA species, beyond 4-bp AS tRNA^Trp^_CCA_. This hypothesis has several theoretical implications.

**Figure 7.**
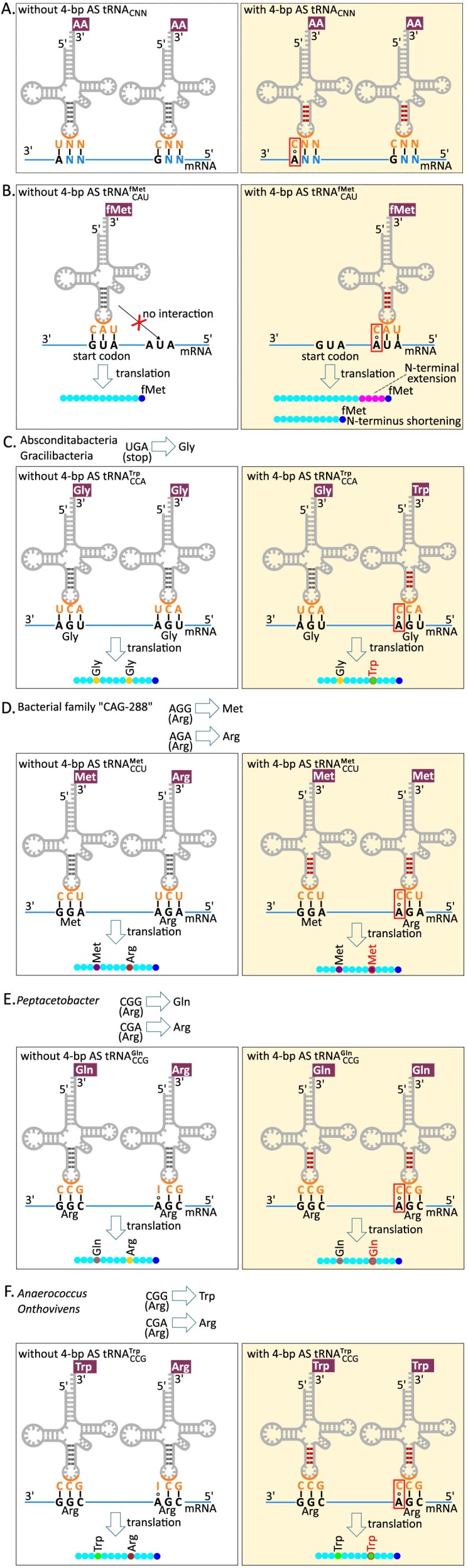
Hypothetical implications of extending the superwobble hypothesis to include C:A base pairing. Panel (A) depicts functionally relevant deployment of 4-bp AS tRNA with a CNN anticodon for decoding NNA codons (note that the codons are depicted in the 3′-to-5′ direction to preserve the pairing anticodon sequences in the conventional 5′-to-3′ direction). Panels (B–F) present situations where the hypothetical ability of a 4-bp AS tRNA with a CNN anticodon to decode NNA codons would lead to detrimental effects and hence selection pressure against the occurrence of the respective 4-bp AS tRNA in the given bacterium. (**A**) Organisms that lack a 4-bp AS tRNA with C at the first anticodon position require the isoacceptor tRNA with U at the first anticodon position to enable efficient decoding of NNA codons (left). Organisms that encode a particular 4-bp AS tRNA with C at the first anticodon position do not need an isoacceptor tRNA with U at this position (right). (**B**) The 5-bp AS tRNA^fMet^_CAU_ recognizes the start codon (AUG) in mRNA sequence and incorporates formylmethionine to a polypetide chain (left). The 4-bp AS tRNA^fMet^_CAU_ could hypothetically recognize AUA triplets up- or downstream of the start codon, which may lead to the production of proteins with an altered N-terminus (extended or shortened; right panel). (**C**) Organisms with stop-to-glycine UGA reassignment (those of the class JAEDAM01, uniting the groups also known as Absconditabacteria and Gracilibacteria) are expected to lack 4-bp AS tRNA^Trp^_CCA_ (left) because such tRNAs could erroneously read UGA codons as tryptophan (right). (**D**) Organisms where arginine AGG codon was reassigned to encode methionine (e.g. in bacteria from the genus *Candidatus* Enterosoma of family CAG-288), while AGA preserves its original meaning and codes for arginine, should lack 4-bp AS tRNA^Met^_CCU_ decoding the AGG codon (left). Otherwise, mistranslation of the AGA as methionine could occur (right). (**E, F**) In organisms that have reassigned the CGG codon from arginine to another amino acid (either glutamine as in *Peptacetobacter*, or tryptophan as in *Anaerococcus* and *Onthovivens*), while decoding CGA as arginine, the tRNA decoding CGG is expected to have a 5-bp AS (left). The presence of 4-bp AS variant could lead to misreading of the CGA codon (right). The situations without and with C:A base pairing are in the boxes with transparent and yellow background, respectively. The tRNA anticodons are highlighted in orange; C:A base pairing, as assumed in the extended superwobble hypothesis, is shown with red rectangles. Watson–Crick base pairs and wobble interactions are shown as dashes and circles, respectively. The amino acids erroneously included in the polypeptide chain as a consequence of C:A base pairing are highlighted with red contour lines. Codon reassignments assumed in a particular case are shown at the top of each panel.

First, assuming the extended superwobble hypothesis is valid, bacteria that encode a particular 4-bp AS tRNA with a C at the first anticodon position do not need an isoacceptor tRNA with a U at the first anticodon position, whereas bacteria that only encode the 5-bp AS variant require such an isoacceptor tRNA to enable efficient decoding of NNA codons (Fig. 7A). We analysed tRNAs for the 11 amino acids that can be encoded by both NNG and NNA codons and decoded by tRNAs with the CNN and UNN anticodons, respectively (Supplementary Fig. S7). For 5 *tRNA_CNN_* species, the fraction of bacterial genomes encoding *tRNA_UNN_* in the presence of 4-bp AS *tRNA_CNN_* was significantly lower compared to the proportion of genomes encoding solely 5-bp AS, as predicted by the extended superwobble hypothesis. However, in four *tRNA_CNN_* cases, the opposite trend was observed, and in the three remaining cases, no statistically significant differences were detected (Supplementary Fig. S7).

Second, the 4-bp AS formylmethionine tRNA (tRNA^fMet^_CAU_) is predicted to be eliminated by selection because it could cause production of proteins with potentially deleterious alterations of the N-terminal regions of proteins (Fig. 7B). The initiator *tRNA^fMet^_CAU_* is the 5th most abundant tRNA in the bacterial dataset (Fig. 1). However, the proportion of 4-bp AS *tRNA^fMet^_CAU_* is extremely low (82 out of the 50 849 identified *tRNA^fMet^_CAU_* in bacteria, or 0.2%), with more than half of these (46 out of 82) having the AS unpinned at the top. For comparison, the percentage of 4-bp AS tRNAs for genes of similar abundance is 14.9% for *tRNA^Asn^_GUU_* (4th most abundant tRNA in the bacterial dataset) and 11% for *tRNA^Lys^_UUU_* (6th most abundant tRNA) (Fig. 1). Thus, 4-bp AS *tRNA^fMet^_CAU_* indeed appears to be eliminated by purifying selection. Although this observation is compatible with our hypothesis, the selection pressure against 4-bp AS tRNA^fMet^_CAU_ might not be caused solely by its undesired putative propensity to decode AUA codons as methionine. It is highly likely that the major factor constraining the features of tRNA^fMet^_CAU_ is its binding to the P site and not the A site of the ribosome, in which the AS plays an important role, contributing to accurate translation initiation [91, 92]. Consequently, any structural alteration of tRNA^fMet^_CAU_, including changes in the number of base pairs in its AS, might be selected against to preserve efficient and error-free translation initiation. Furthermore, in reality tRNA^fMet^_CAU_ is expected to be capable of binding non-AUG codons, as translation initiation in bacteria quite frequently starts at codons other than AUG [93]; the NCBI translation table 11 used for annotation of bacterial genomes even allows AUA (i.e. the codon predicted to be recognized by a 4-bp AS tRNA^fMet^_CAU_ under the superwobble hypothesis) as an initiation codon.

In the case of the elongator tRNA^Met^_CAU_, which binds to the A site of the ribosome, the 4-bp AS variant represents 27.3% of all identified *tRNA^Met^_CAU_* (11 356 out of 41 644) genes in bacteria, of which 2272 (~20.0%) have AS unpinned at the top. It appears that bacteria are quite tolerant to the presence of 4-bp AS elongator tRNA^Met^_CAU_ given that misincorporation of methionine at the AUA codons (isoleucine) in the internal position of protein-coding regions is unlikely to cause substantial deleterious consequences [94]. Moreover, assuming that the levels of relative incorporation of isoleucine versus methionine at the AUA codons is regulated (e.g. via changing the relative abundance of 4-bp AS elongator tRNA^Met^_CAU_ and the natural AUA-cognate tRNA), decoding of the AUA codon as methionine might provide a protective mechanism for coping with oxidative stress because many oxidants react with methionine residues to form methionine sulfoxide, which can be reduced back to methionine by methionine sulfoxide reductases [95].

Third, genomes with stop-to-glycine UGA reassignment are expected to lack 4-bp AS tRNA^Trp^_CCA_ because it could misread the UGA codon as tryptophan (Fig. 7C). In our dataset, 53 bacterial genomes representing the class JAEDAM01 in the phylum Patescibacteriota demonstrate the stop-to-glycine UGA reassignment (Supplementary Table S1) as reported previously [44, 96, 97]. Among these, only four genomes (three forming a clade and one separate) were found to encode a 4-bp AS tRNA^Trp^_CCA_ variant. All four genomes encode exclusively the 4-bp AS tRNA^Trp^_CCA_ variant. Of note, three of these differ from the *Blastocrithidia*-like tRNA in the D-loop length, and one carries a G24A mutation; therefore, experimental validation is required to confirm that these variants decode UGA as tryptophan. In the 43 genomes, only the 5-bp AS variant is present, while in the six remaining genomes, we were unable to identify a *tRNA^Trp^_CCA_* gene likely due to incomplete genome assembly.

Fourth, bacterial genomes, in which AGG and AGA code for different amino acids (methionine and arginine, respectively), should be depleted for 4-bp AS tRNA decoding the AGG codon to avoid misreading the AGA codon (Fig. 7D). In the analysed dataset, three genomes from the genus *Candidatus* Enterosoma of the family CAG-288 demonstrate the arginine-to-methionine AGG reassignment. Additionally, we included six genomes from the same bacterial group reported to have the same codon reassignment that are hypothesized to employ tRNA^Arg^_CCU_ with methionine tRNA identity elements to decode the AGG codon as methionine [44]. Indeed, these 9 genomes encode exclusively 5-bp AS tRNA_CCU_ with methionine identity elements. Additionally, we examined tRNAs in 19 other genomes from the family CAG-288 that do not exhibit signs of the arginine-to-methionine AGG reassignment. Among these, we identified tRNA^Arg^_CCU_ genes in six genomes, all of these with the arginine identity elements and exclusively 5-bp AS. Because only 5-bp AS tRNA^Arg^_CCU_ are found in the subset of bacteria with the standard genetic code, it remains unclear whether the absence of 4-bp AS tRNA^Arg^_CCU_ in the group with the AGG reassignment reflects the results of negative selection or is an ancestral feature of this lineage.

Fifth, in the three separate lineages of phylum Bacillota that have reassigned the CGG codon from arginine to another amino acid (tryptophan or glutamine), while decoding CGA as arginine, the tRNA decoding CGG is expected to have a 5-bp AS to prevent misreading of the CGA codon (Fig. 7E and F). In contrast, this constraint should not apply to bacteria that interpret both CGG and CGA as the same amino acid. Indeed, in the two *Peptacetobacter* genomes, which evolved the arginine-to-glutamine CGG reassignment, tRNA_CCG_ has a canonical 5-bp AS, but the situation is more complex in the other two lineages (the unrelated genera *Anaerococcus* and *Onthovivens*). Among the 17 genomes with predicted arginine-to-tryptophan CGG reassignment, 14 contained an identifiable tRNA_CCG_ gene. Contrary to our prediction, eight of these genomes (1 and 7 in the genera *Onthovivens* and 7 *Anaerococcus*, respectively) encode a 4-bp AS tRNA_CCG_ (Supplementary Table S3). Furthermore, in Absconditabacterales that decode both CGG and CGA as tryptophan rather than the standard arginine and hence should tolerate 4-bp AS tRNA_CCG_, all tRNAs with the CCG anticodon have a 5-bp AS (Supplementary Table S3).

Thus, the occurrence of 4-bp AS tRNAs is subject to evolutionary constraints depending on the functional context. Although some of the results above further support, albeit indirectly, the extended superwobble hypothesis, experimental evidence is needed to confirm the substantial expansion of tRNA decoding capabilities beyond *Blastocrithidia-*like tRNA^Trp^_CCA_.

## Supplementary Material

gkag327_Supplemental_Files

## Data Availability

The data underlying this article are available in the article and in its online supplementary material. The input data for reproducing the MD simulations, raw results files, and scripts needed for generating Fig. 6 are available at https://github.com/kolarlab/fakih-4bp-trnas and https://doi.org/10.5281/zenodo.19220793.

## Notes

Present address: Department of Biochemistry, University of Cambridge, CB21GA Cambridge, United Kingdom

Present address: Department of Zoology, Banaras Hindu University, Varanasi 221005, India
