# Supplementary material for: Frequent occurrence and predicted functions of tRNAs with 4-base-pair anticodon stems in bacteria: extended superwobble hypothesis
Source: Nucleic Acids Res. 2026 Apr 20;54(7):gkag327. doi: 10.1093/nar/gkag327 (PMC13092985; doi:10.1093/nar/gkag327)
Supplement: gkag327_Supplemental_Files [file gkag327_supplemental_files.zip › Supplementary_figures.pdf]

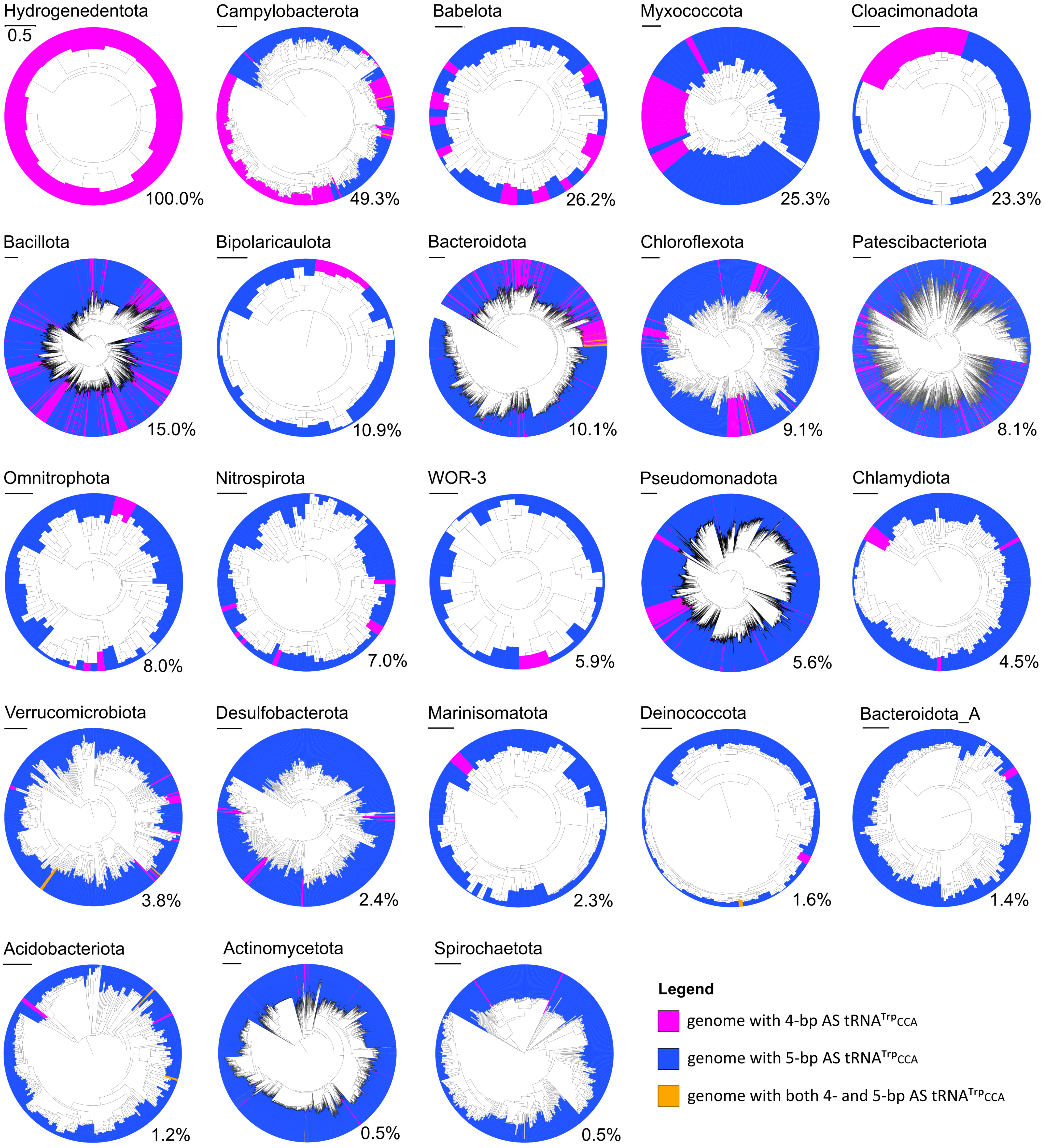

Suppl. fig. 1. Distribution of 4- and 5-bp $tRNA^{Trp}CCA$ variants across bacterial phyla. Visualisation of the bacterial phylogenomic tree from the Genome Taxonomy Database (GTDB) release 214.1 depicting evolutionary relationships within phyla that have at least 10 representatives in the bacterial dataset and include a minimum of two species encoding 4-bp AS $tRNA^{Trp}CCA$. Taxonomic information was updated according to the GTDB release 226. Within each phylum, only species possessing at least one *$tRNA^{Trp}CCA$* gene are shown. Species encoding exclusively 4-, 5-bp, or both $tRNA^{Trp}CCA$ variants are highlighted in magenta, blue, and orange, respectively. Horizontal line represents 0.5 substitutions per site. Percentages indicate the proportion of species containing the 4-bp variant relative to all species shown in the tree.

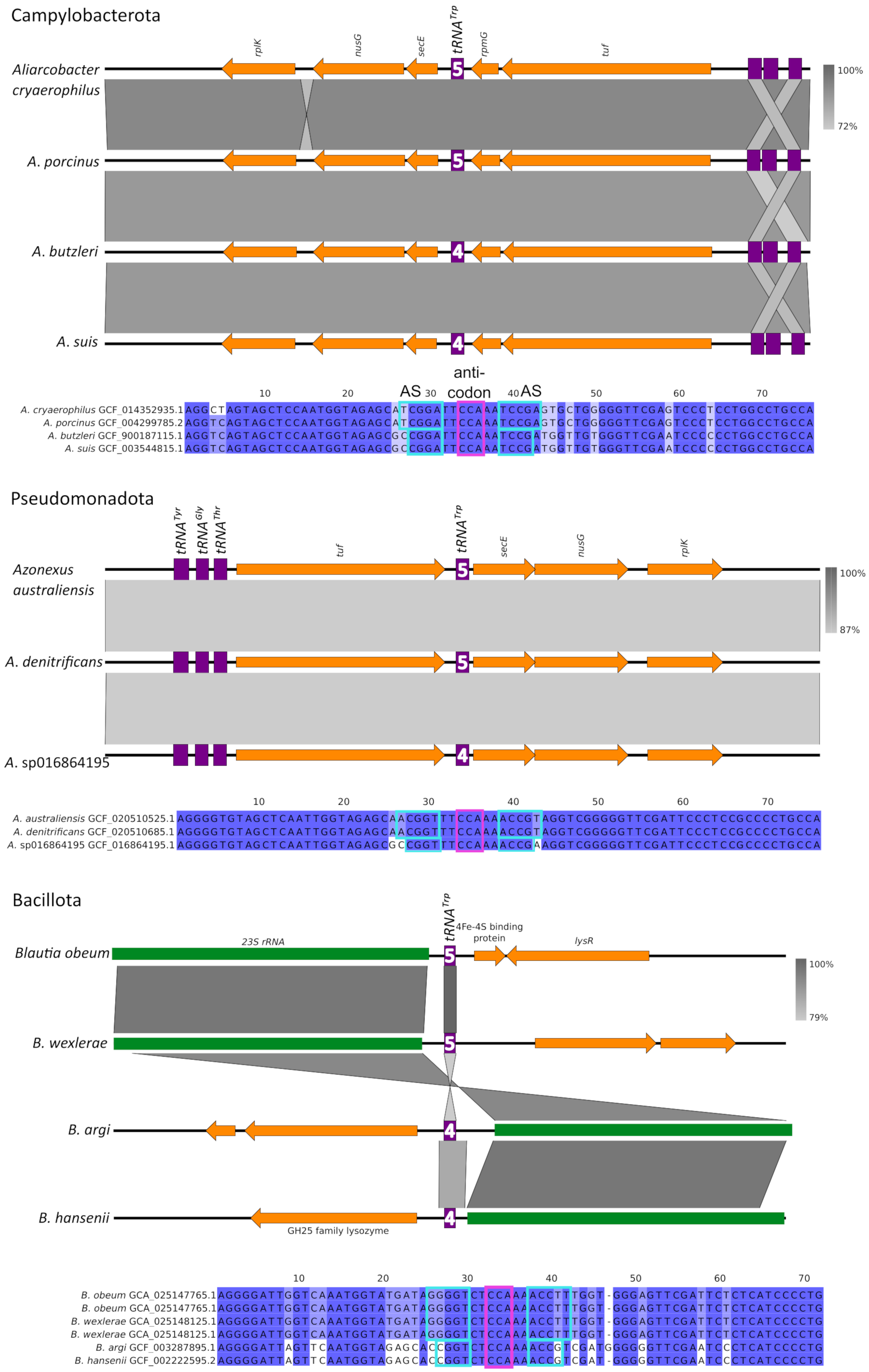

Suppl. fig. 2. Synteny analysis. A schematic representation of gene synteny within a 4,000-nt region surrounding the *tRNA*^{Trp}*CCA* locus is shown for three bacterial genera: *Aliarcobacter*, *Azonexus*, and *Blautia*. tRNA, rRNA, and protein-coding genes are shown as violet, green, and orange rectangles, respectively. For tRNA^{Trp}CCA, the number of paired bases within the AS is indicated with a number. Syntenic regions are connected by grey rectangles, with darker shades indicating higher sequence identity, as per colour legend. Below each synteny graph, an alignment of tRNA^{Trp}CCA is displayed with shading intensity corresponding to sequence identity levels (darker blue represents higher identity). Nucleotides of the predicted anticodon stem (AS) and anticodon are boxed. Products of protein-coding genes: *lysR* - lysine regulator; *nusG* - N-utilization substance G; *rplK* - large ribosomal subunit protein uL11; r*pmG* - large ribosomal subunit protein bL33; *secE* - secretion protein E; *tufA* - elongation factor Tu.

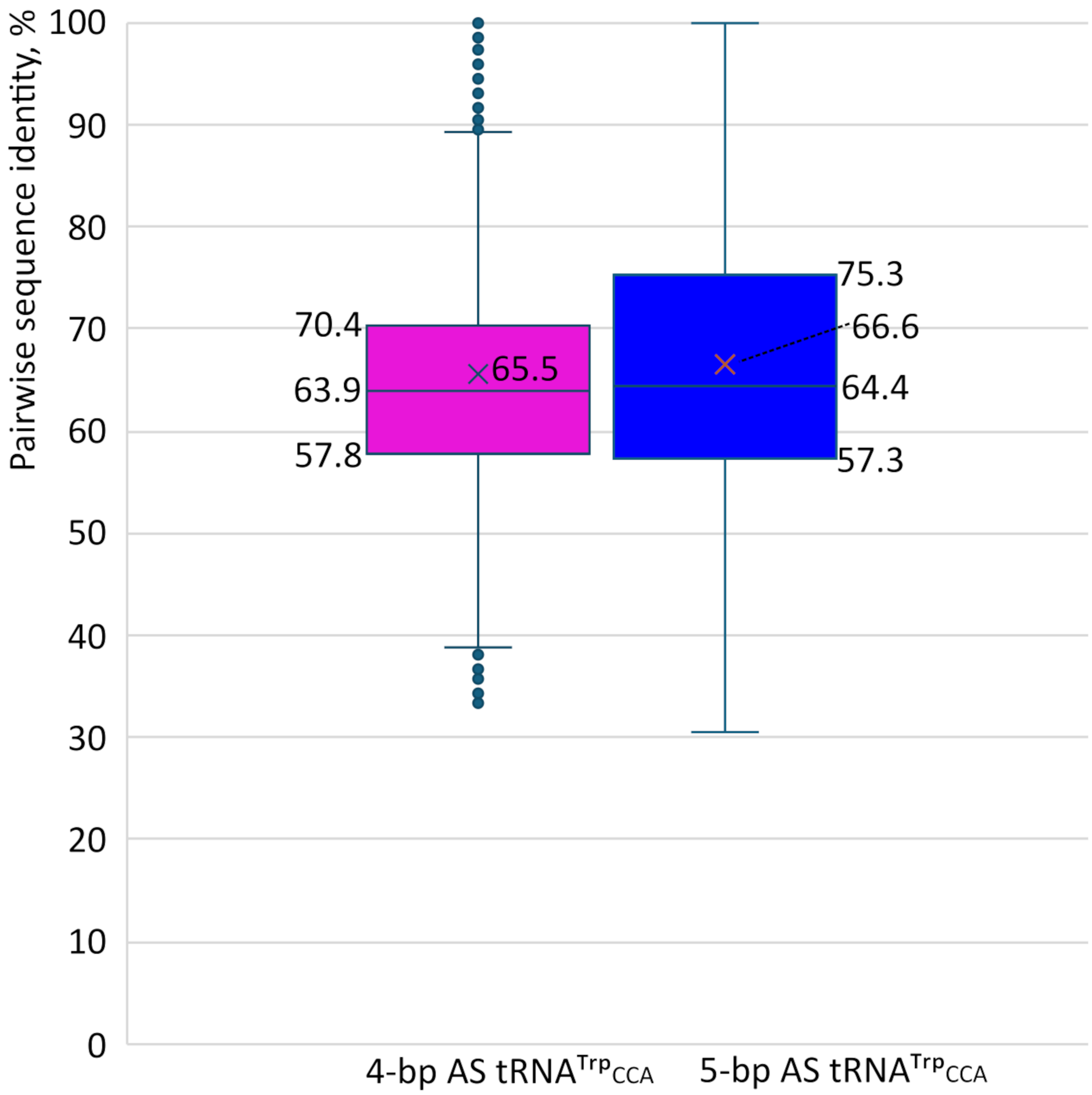

Suppl. fig. 3. Boxplot representation of pairwise identity values among 4- and 5-bp AS $tRNA^{Trp}_{CCA}$. The data is derived only from those bacterial genomes where 4- and 5-bp AS molecules co-occur. The lower and upper edges of the box represent the lower and upper quartiles, respectively; the horizontal line inside the box denotes the median value; the cross indicates mean value.

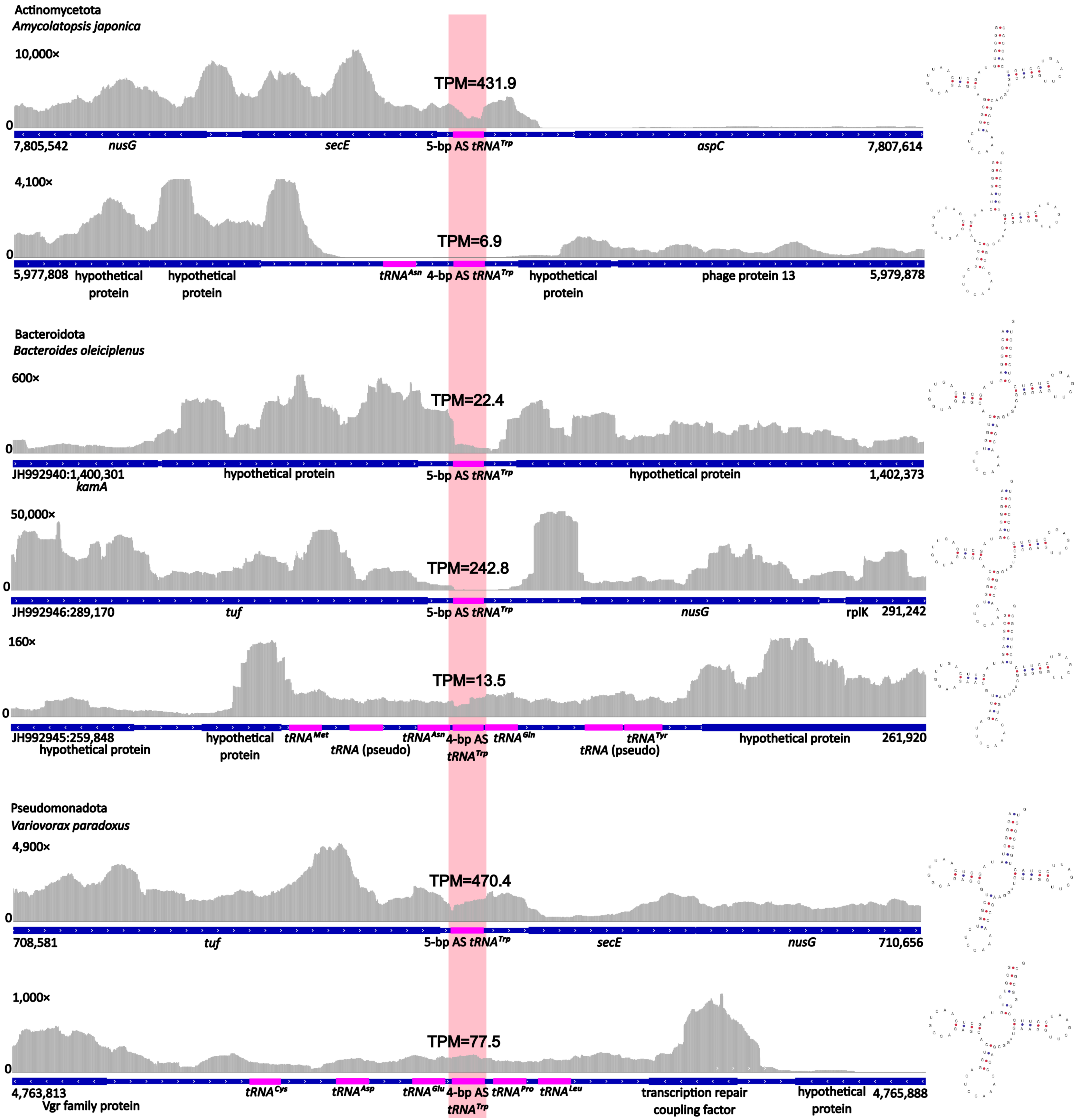

Suppl. fig. 4. *tRNA^{Trp}CCA* with 4- and 5-bp AS rarely co-occur in the same bacterial genomes. A snapshot of RNA-seq data mapping onto the genome assembly at the *tRNA^{Trp}CCA* loci (highlighted in light pink) and 1,000 nt up- and downstream in various bacteria. Co-ordinates of the displayed region in the genome assembly are indicated (scaffold names are shown only when the assembly contains multiple scaffolds). Protein-coding and tRNA genes are depicted by blue and magenta rectangles, respectively. The upper track shows RNA-seq read coverage (with the maximum value indicated on Y axis). Nucleotide positions in the reads different from the reference and supported by the read fraction ≥0.3 are highlighted in the coverage track: A – green, T – red, G – blue, C - orange. Transcriptomic data from public databases were used to generate coverage plots (see Materials and Methods for details). Predicted tRNA secondary structures are depicted on the right. Products of protein-coding genes: *aspC* - aspartate aminotransferase; *kamA* - L-lysine 2,3-aminomutase; *nusG* - N-utilization substance G; *rplK* - large ribosomal subunit protein uL11; *tufA* - elongation factor Tu 1; *Vgr* - valine-glycine repeat. Abbreviations: TMP - transcripts per million.

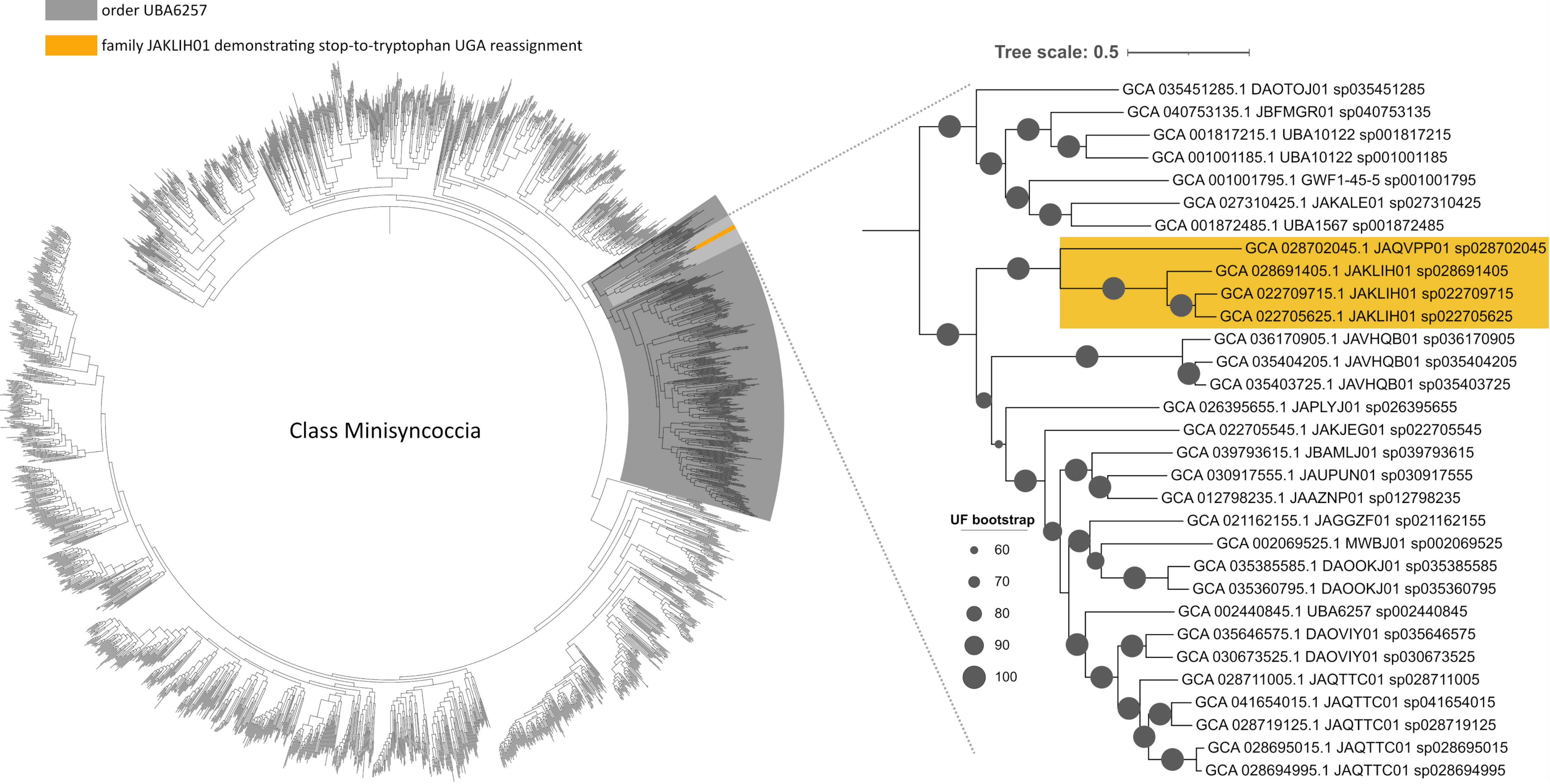

Suppl. fig. 5. Phylogenetic position of the bacterial family JAKLIH01 with a newly reported case of a stop-to-tryptophan UGA reassignment in the phylum Patescibacteriota. Phylogenomic tree of the class Minisyncoccia from the GTDB release 226 (left) shows the placement of the family JAKLIH01 within the order UBA6257. The phylogenomic tree inferred in this study (right), based on 71 proteins encoded by single-copy genes present in at least 80% of the analysed species, illustrates the relationships among representatives of JAKLIH01 and their closest relatives. The monophyly of the family JAKLIH01 supports a single evolutionary origin of the stop-to-tryptophan UGA reassignment in this group. The tree scale shows the number of substitutions per site. UF – ultrafast bootstrap support.

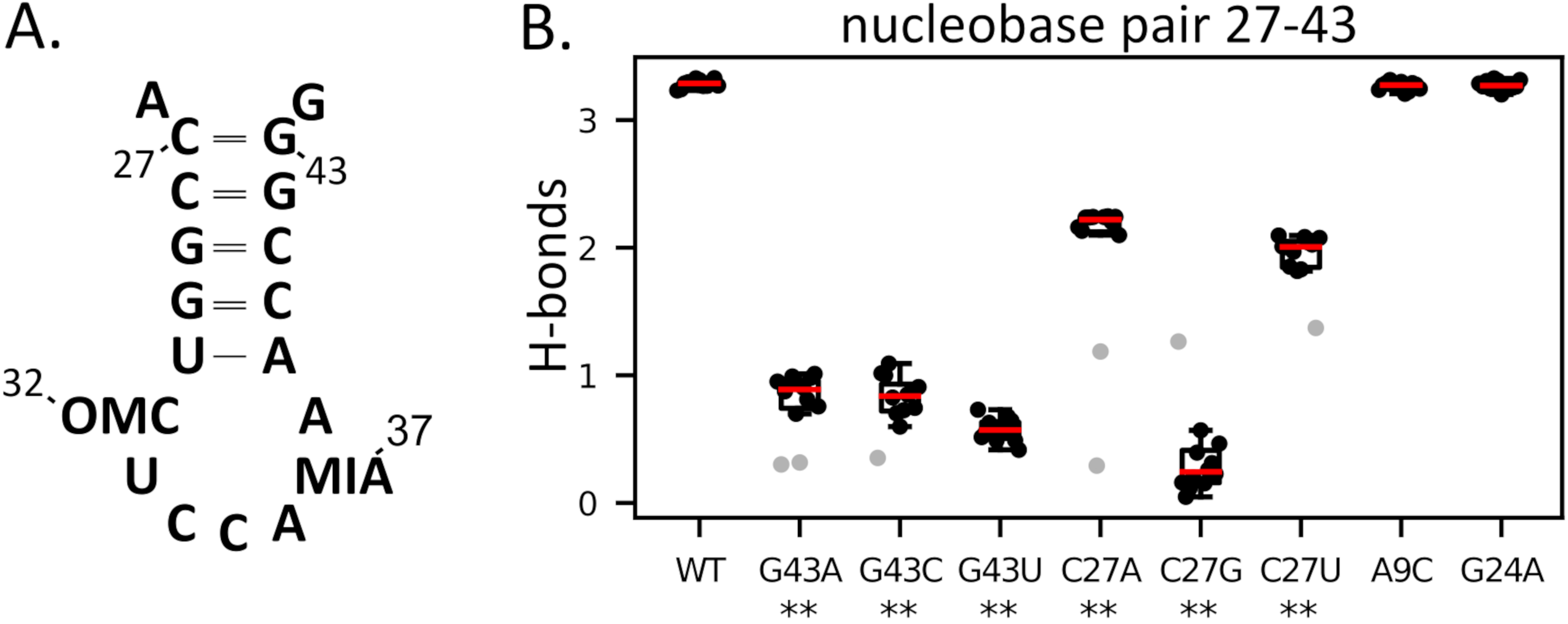

Suppl. fig. 6. Atomistic molecular dynamics simulations of the hydrogen bonds (H-bonds) between the top nucleobases within the AS of the *Escherichia coli* tRNA^Trp^CCA. A. 2D depiction of the anticodon region of the ***E. coli*** tRNA^{Trp}CCA. B. Number of H-bonds between the nucleobases 27 and 43 within the anticodon stem of several 4-bp *Blastocrithidia*-like tRNA^{Trp}CCA variants compared to the 5-bp AS wild-type, A9C and Hirch G24A variants. The boxes indicate the interquartile range, the red line denotes the median, whiskers extend to the most extreme data points within 1.5× the interquartile range, and grey circles represent outliers. Mann-Whitney U-test statistics were calculated from 12 values, each obtained as the average over one of the 12 independent trajectories. $^{**}p < 0.01$.

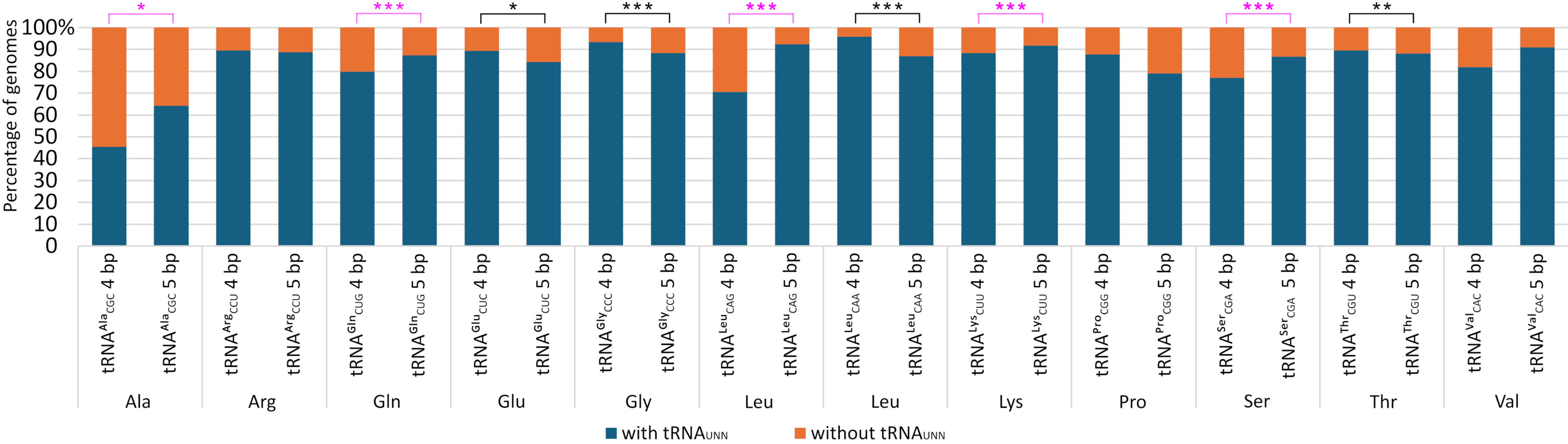

Suppl. fig. 7. Barplots demonstrating the occurrence of tRNAUNN in the bacterial genomes along with the 4-bp AS tRNACNN or in the absence of it. The proportion of bacterial genomes possessing tRNA**UNN** along with the 4-bp AS tRNACNN or in the absence of it (in the genomes possessing solely 5-bp AS tRNACNN) is shown on Y axis; X axis shows all amino acids, which can be encoded by NNA and NNG codons. The proportion of the genomes possessing and lacking tRNAUNN are shown in blue and orange, respectively. Statistical significance levels for Z-test: * - *p*-value <0.05; ** - *p*-value <0.01; *** - *p*-value <0.001. The cases where a tRNAUNN occur in the presence of a 4-bp AS tRNACNN significantly less frequently than in the background of a 5-bp AS tRNACNN are shown with magenta lines above the barplot.
